# Gut microbiota in irritable bowel syndrome: a narrative review of mechanisms and microbiome-based therapies

**DOI:** 10.3389/fimmu.2025.1695321

**Published:** 2025-11-06

**Authors:** Xuemei Li, Qiang Yuan, Hui Huang, Li Wang

**Affiliations:** 1 Department of Clinical Medicine, Chengdu Medical College, Chengdu, Sichuan, China; 2 Department of Gastroenterology, First Affiliated Hospital of Chengdu Medical College, Chengdu, Sichuan, China

**Keywords:** irritable bowel syndrome, gut microbiota, immune response, pathogenesis, fecal microbiota transplantation

## Abstract

Irritable bowel syndrome (IBS) is a common disorder of gut–brain interaction, and its pathogenesis remains unclear. Dysbiosis of the gut microbiota is associated with IBS. The gut microbiota may modulate IBS symptoms via the epithelial barrier, mucosal immunity, microbial metabolites (e.g., short-chain fatty acids and bile acids), and gut–brain signaling. Currently, dietary approaches, probiotics, prebiotics, rifaximin, and fecal microbiota transplantation show variable benefit; effects are strain-/context-dependent and evidence certainty varies, with adverse-event reporting inconsistent. This narrative review takes a subtype-aware, mechanism-first perspective to summarize microbiota functions, symptom links, and intervention evidence with safety considerations. This review offers new perspectives and insights for precision treatment and microbiome research in IBS.

## 1 Introduction

**Figure 1:**
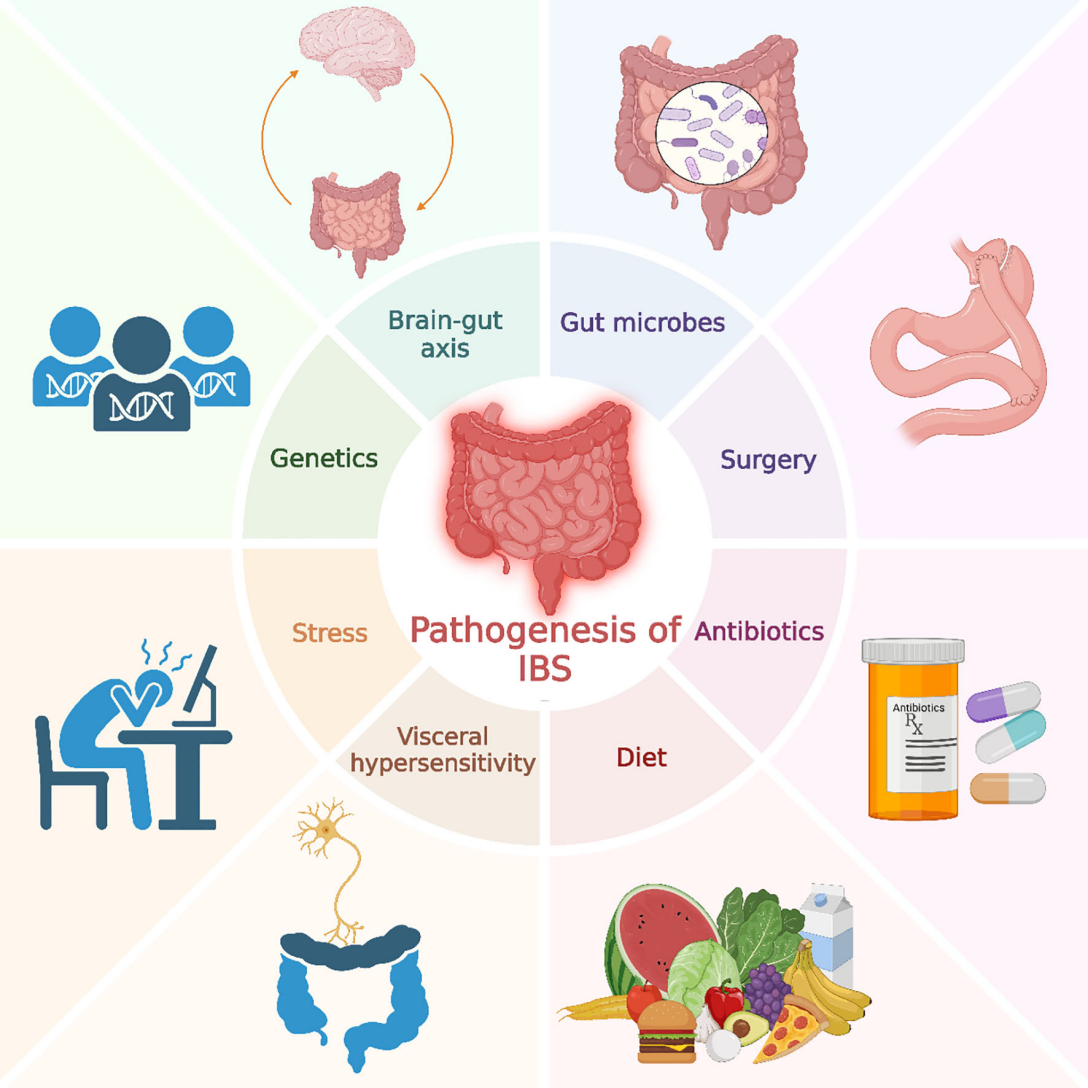
Pathogenesis of irritable bowel syndrome. This figure illustrates various factors contributing to the pathogenesis of IBS, including genetics, stress, gut microbes, diet, antibiotics, surgery, visceral hypersensitivity, and the brain-gut axis.

Irritable bowel syndrome (IBS) is a chronic disorder of gut-brain interaction, characterized by recurrent abdominal pain associated with changes in stool frequency or form (1). According to the latest global study by the ROME Foundation conducted in 33 countries, the global prevalence of IBS is estimated at 3% to 5% (2). Based on the Rome IV criteria, IBS can be classified into four types based on the predominant stool pattern: IBS-D (diarrhea-predominant), IBS-C (constipation-predominant), IBS-M (mixed type), and IBS-U (unclassified) (3). Despite the absence of identifiable organic lesions in the intestines, IBS significantly impacts patients’ quality of life and places a substantial burden on healthcare systems and society. The pathophysiology of IBS is recognized as multifactorial, although the exact mechanisms remain unclear (4). **Figure 1** summarizes this multifactorial model: genetic susceptibility establishes host predisposition; psychosocial stress and autonomic dysregulation modulate motility, pain processing and immune function; microbial dysbiosis—exacerbated by antibiotics or surgery—shifts metabolite outputs (e.g., SCFAs, bile acids, gases) and impairs epithelial integrity; diet acts as both a trigger (FODMAPs) and substrate (fiber for SCFAs); and visceral hypersensitivity represents a final common pathway amplifying pain perception. The bidirectional brain–gut axis links these domains, such that changes at one node (e.g., barrier dysfunction) can propagate to others (e.g., immune activation and central sensitization). Emerging evidence suggests that the gut microbiota plays a crucial role in the onset and progression of IBS. The gut microbiota, a critical platform for host-environment interactions, consists of trillions of microorganisms. It not only participates in the host’s digestive processes but also regulates host health and disease states through interactions with the immune, metabolic, and nervous systems (5).

The literature for this review was selected through a comprehensive search of PubMed and Web of Science, primarily focused on literature published between January 2015 and July 2025. The search strategy included “irritable bowel syndrome,” “IBS,” “gut microbiota,” “dysbiosis,” and “microbiome,” as well as combinations of keywords related to interventions such as “probiotics,” “prebiotics,” “FMT,” “diet,” and “antibiotics,” and combinations of keywords related to mechanisms such as “gut-brain axis,” “visceral hypersensitivity,” “intestinal permeability,” “immune activation,” “stress,” and “gut microbial metabolites.” Our inclusion criteria prioritized: (1) high-impact studies such as systematic reviews, meta-analyses, and large, well-designed randomized controlled trials (RCTs); (2) original research articles elucidating key pathophysiological mechanisms (e.g., gut-brain axis, immune activation, barrier function); (3) studies covering the main IBS subtypes (IBS-D, IBS-C, IBS-M); and (4) foundational papers that are widely cited for establishing key concepts. Exclusion criteria included: (1) case reports, small uncontrolled case series, and abstracts without full-text availability; (2) studies with significant methodological limitations or a high risk of bias; and (3) non-peer-reviewed articles, editorials, and opinion pieces (unless providing a unique, widely accepted perspective).

Many studies show that IBS patients often exhibit dysbiosis, which is characterized by a decrease in gut microbiota diversity and an abnormal relative abundance of specific microbial groups (6). Changes in the composition of the gut microbiota are closely associated with IBS clinical symptoms, impaired gut barrier function, and immune system abnormalities. Metabolites of the gut microbiota, such as short-chain fatty acids (SCFAs) and bile acids, are linked to epithelial barrier, mucosal immune, and gut–brain signaling pathways relevant to IBS (7). The gut-brain axis and visceral hypersensitivity are also recognized as key factors influencing IBS symptoms (8). Multiple high-quality reviews have summarized microbiome alterations in IBS. However, most either emphasize global dysbiosis without integrating subtype-specific mechanisms, or remain taxonomy-centric with limited linkage from microbial functional outputs (e.g., short-chain fatty acids, bile acids, microbial gases, tryptophan-derived metabolites) to host pathways (barrier integrity, mucosal immunity, enteric neurotransmission, and gut–brain signaling) and symptom generation. Intervention-focused narratives commonly consider single modalities (e.g., diet, probiotics, or fecal microbiota transplantation) rather than comparing modalities within a unified framework that grades evidence certainty and addresses safety.

To complement prior reviews and address these gaps, this review offers a distinct, mechanism-first synthesis. Our primary novelty lies in three areas. First, we move beyond a purely taxonomic description to a function-oriented view, linking key microbial outputs (SCFAs, bile acids, gases, and tryptophan metabolites) directly to the host pathophysiological pathways they modulate across the epithelial, immune, and neural systems. Second, we adopt a subtype-aware approach, systematically connecting these functional mechanisms to the distinct symptom profiles of IBS-D, IBS-C, and IBS-M. Third, we provide an integrated evidence map of major microbiome-based interventions—from diet and probiotics to rifaximin and fecal microbiota transplantation (FMT)—that compares their efficacy, summarizes safety considerations, and appraises the certainty of evidence within a single, comparative framework. This review provides new insights for the precision treatment of IBS and offers a conceptual foundation for further development in the field of gut microbiome research.

## 2 Gut microbiota and IBS

The gut microbiota refers to the entire microbial community residing in the host’s intestines, a complex ecosystem consisting of trillions of microorganisms, including bacteria, fungi, archaea, and viruses. The gut microbiota is not merely a passive participant in the host’s digestive processes, but plays a crucial regulatory role in host health, immunity, metabolism, and behavior (9). The functions of the gut microbiota extend far beyond traditional digestion. Studies have shown that the gut microbiota interacts with the host’s nervous, immune, and endocrine systems through the gut-brain axis, affecting host behavior, mood, and immune responses (10). The composition of the gut microbiota is dynamic and regulated by various factors such as host age, diet, genetics, and environment (11). In a healthy gut microbiota, several microbial groups predominate. Research indicates that in healthy individuals, the gut microbiota is composed of approximately 40%-60% Firmicutes, 30%-40% Bacteroidetes, and 5%-10% Actinobacteria, while the proportion of Proteobacteria is relatively low (<5%) (12). These dominant microbial communities work together to maintain microbial balance and promote intestinal function. Among these dominant microbial groups, certain bacterial genera such as *Bifidobacterium*, *Lactobacillus*, *Faecalibacterium*, and *Lactococcus* are abundant and considered crucial for maintaining gut health (13). These commensal bacteria interact with the host’s immune system and intestinal epithelial cells, playing a role in regulating gut immunity, maintaining the intestinal barrier function, and inhibiting the growth of harmful pathogens. However, when the balance of the gut microbiota is disrupted, this dysbiosis may lead to various gastrointestinal diseases. Dysbiosis, characterized by a reduction in microbial diversity and the overgrowth of certain harmful bacteria, has been implicated in numerous gastrointestinal disorders, including IBS.

Recent studies have shown that the development of IBS is closely related to dysbiosis of the gut microbiota (**Table 1**, **2**). In IBS patients, a hallmark feature is reduced microbial diversity and changes in the relative abundance of specific bacterial groups, which are associated with increased severity of IBS symptoms (6). Firmicutes and Bacteroidetes are the major components of the gut microbiota in healthy adults, and the ratio between these two phyla is considered an important indicator of gut microbial balance (14). A meta-analysis of 16 studies involving 777 IBS patients and 461 healthy controls found that, at the phylum level, IBS patients showed an increased Firmicutes-to-Bacteroidetes ratio, indicating dysbiosis. At lower taxonomic levels, an increase in *Clostridium* and *Clostridiales* was observed, while Bacteroides and *Bacteroidales* were reduced (15). This imbalance was more pronounced in IBS-D patients. The decrease in Bacteroidetes may lead to reduced intestinal anti-inflammatory capacity, while the excessive increase in Firmicutes could be associated with intestinal inflammation and worsened symptoms. Another meta-analysis comprising 23 studies and 1,340 participants indicated that compared to healthy controls, IBS patients had lower levels of *Lactobacillus* and *Bifidobacterium* in their stool samples, while *Escherichia coli* and *Enterococcus* levels were higher (16).

**Table 1:** Summary of studies on IBS and gut microbiota dysbiosis.

Author (year)	Country	Study type	Patient sample size	Method	Sample type	Rome criteria	Main results
Dlugosz et al. (2015) (196)	Sweden	RCT	35	qPCR	Jejunal mucosa	Rome II	No differences
Pozuelo et al. (2015) (197)	Europe	RCT	113	16S rRNA	Feces	Rome III	Reduced microbial diversity and decreased butyrate-producing bacteria in IBS-D and IBS-M patients
Shukla et al. (2015) (17)	India	RCT	47	16S rRNA	Feces	Rome III	Decreased *Lactobacillus* in IBS-D compared to IBS-C, with higher *Pseudomonas* and Bacteroides in both subtypes
Tap et al. (2017) (18)	Sweden	RCT	110	16S rRNA	Feces and mucosa	Rome III	No difference in α or β diversity between IBS and healthy controls, Bacteroides increased, *Prevotella* and *Methanobrevibacter* decreased in IBS
Liu et al. (2017) (198)	China	Systematic review and meta-analysis	360 (13 studies)	qPCR	Feces and mucosa	Rome II & III	Significant differences in *Lactobacillus*, *Bifidobacterium*, and *Faecalibacterium* expression in IBS patients compared to controls
Zhong et al. (2019) (19)	China	RCT	20	FISH	Rectal and colon mucosa	Rome III	Increased *Escherichia coli*, *Clostridium*, and Bacteroides in IBS-D, with decreased *Bifidobacterium* and negative correlation with stool frequency
Jeffery et al. (2020) (28)	Ireland	RCT	80	16S rRNA	Feces	Rome IV	Increased *Ruminococcus gnavus* and *Lachnospiraceae* and decreased *Barnesiella intestinihominis* and *Coprococcus catus* in IBS
Wang et al. (2020) (16)	USA	Systematic review and meta-analysis	1,340 (23 studies)	16S rRNA	Feces	Rome IV	IBS patients showed lower *Lactobacillus* and *Bifidobacterium* and higher *Escherichia coli* compared to healthy controls
Jacobs et al. (2023) (33)	USA	Cross-sectional cohort study (multi-omics)	495	Multi-omics: 16S rRNA sequencing, metatranscriptomics & metabolomics	Feces	Rome IV	IBS is characterized by an increased abundance of *Alistipes ihumii, Bacteroides dorei, Actinomyces odontolyticus*, as well as several members of the phylum Firmicutes (such as *Intestinibacter bartlettii* and *Romboutsia ilealis*), and a decreased abundance of *Faecalibacterium prausnitzii* and *Bacteroides thetaiotaomicron*.
Li et al. (2024) (199)	China	Systematic review and meta-analysis	1167 (7 studies)	16S rRNA	Feces	Rome III & IV	Patients with IBS exhibit an increased abundance of Ruminococcaceae, *Anaerostipes*, and Christensenellaceae.
Sánchez-Pellicer et al. (2025) (26)	Spain	Case–control study	135	16S rRNA	Feces	Rome IV	In IBS, Bacteroides increases, while *Agathobacter*, *Subdoligranulum*, and the *Christensenellaceae* R-7 group decrease.

IBS, irritable bowel syndrome; IBS-D, diarrhea-predominant irritable bowel syndrome; IBS-C, constipation-predominant irritable bowel syndrome; IBS-M, mixed-type irritable bowel syndrome; RCT, randomized controlled trial; qPCR, quantitative polymerase chain reaction; 16S rRNA, 16S ribosomal RNA (amplicon sequencing); FISH, fluorescence *in situ* hybridization.

**Table 2:** Structured taxonomy & functional roles in IBS.

Phylum → genus/species	Putative role in IBS	Notes	Reference
Firmicutes → *Faecalibacterium prausnitzii*	Anti-inflammatory; butyrate-producer	Often reduced; barrier relevance	(23, 200)
Firmicutes → *Clostridium cluster XIVa*	Bile-acid metabolism	Enrichment in some IBS-D contexts	(201, 202)
Bacteroidetes → *Bacteroides* spp.	Carbohydrate fermentation	Function > taxonomy across cohorts	(23)
Actinobacteria → *Bifidobacterium* spp.	SCFA production; barrier support	Strain-dependent RCT benefits	(29)
Proteobacteria → *Escherichia*/*Shigella*	Pro-inflammatory/pathobiont	Frequently enriched (heterogeneous)	(36, 203)
Archaea → *Methanobrevibacter smithii*	Methane production; slowed transit	Linked to IBS-C and constipation traits	(60, 204)

However, there are some inconsistencies in the research findings regarding the microbiome characteristics of IBS patients (15). In IBS-D patients, there are conflicting data regarding Actinobacteria and *Bifidobacteria*. Some studies indicate that the abundance of Actinobacteria in the fecal and mucosal samples from IBS-D patients is significantly reduced (17, 18). Zhong et al.’s study showed that *Bifidobacteria*, especially fecal *Bifidobacteria*, were significantly reduced in the mucosal microbiome of these patients (19). However, in contrast to this evidence, two studies have shown that the abundance of Actinobacteria in the fecal microbiome of IBS-D patients is higher (18, 20). Additionally, there are significant differences in the abundance of *Lactobacilli* between IBS patients and healthy controls, but the conclusions of different studies are inconsistent. Some authors report an increase in *Lactobacilli* numbers (21–23), while others observe a decrease in the abundance of this commensal bacterium (16, 24, 25). To illustrate this complexity, a 2025 case-control study comparing 25 IBS patients with 110 healthy individuals found that the IBS microbiota was more “enriched” but had lower α-diversity, accompanied by a decrease in Firmicutes (especially Clostridia) and an increase in Bacteroidota (particularly the family Bacteroidaceae) (26). Through differential analysis, the study proposed Bacteroides, *Faecalibacterium*, and *Blautia* as potential diagnostic biomarkers and highlighted the features of “simplification” and “imbalance” in the IBS microbiome. To place these microbial changes in a broader context, it is useful to compare them with those in inflammatory bowel disease (IBD). A comparative study assessed the mucosa-associated microbiota in 20 patients with IBS-D and 28 patients with UC using fluorescence *in situ* hybridization (FISH) (19). The results revealed that on the mucosal surface and in the mucus layer of both IBS-D and UC patients, the numbers of *E. coli*, *Clostridium*, and Bacteroides were significantly increased, while *Bifidobacterium* was significantly reduced. However, active UC was also characterized by the invasion of the lamina propria by *E. coli* and Bacteroides. Furthermore, bacterial numbers fluctuated more dramatically in UC patients (1.3–5.3 fold), and a reduction in *Lactobacillus* was observed only in UC. These findings suggest that while both IBS and UC share features of dysbiosis, the microecological disruption and bacterial translocation are more pronounced in UC.

Although studies have demonstrated clear differences in the composition and diversity of the microbiota between IBS patients and healthy controls, most studies have failed to detect significant differences when comparing different IBS subtypes (27, 28). However, a 2024 study on constipation-predominant and mixed-type IBS subtypes found that these patients had an increased Firmicutes/Bacteroidetes ratio, an increase in Actinobacteria and Verrucomicrobiota, and a decrease in Bacteroidota. The study also noted that *Anaerostipes hadrus* (a facultative butyrate producer) and *Bacteroides plebeius* were significantly enriched in both subtypes (29). These results highlight the impact of subtype and geographical differences on microbiota structure, suggesting that future intervention strategies need to consider individualized and multidimensional factors.

The inconsistency of findings across IBS microbiome studies can largely be attributed to the combined effects of technical and design-related factors. First, sampling strategies differ substantially and are a fundamental source of discordance. Most studies analyze noninvasive fecal specimens, which represent the luminal community but are highly sensitive to recent diet and intestinal transit time, potentially masking stable, host-interactive microbial features; by contrast, mucosal biopsies, though invasive, capture host-adherent microbes at the epithelial interface where barrier- and immunity-related host–microbe interactions occur. These two ecological niches harbor distinct microbial profiles, meaning discoveries in stool may be absent at the mucosa and vice versa; direct comparison across sample types is therefore problematic and often yields parallel, non-integrable bodies of literature (30). Second, variability extends through laboratory and computational workflows and introduces substantial noise that can be mistaken for biology: from storage and transport (fresh, −80 °C frozen, lyophilized/stabilized) to freeze–thaw cycles, conditions can shift observed diversity and relative-abundance profiles; DNA extraction protocols (lysis intensity/need for mechanical disruption, kit choice) can systematically under-represent Gram-positive taxa and produce high-magnitude, method-dependent differences in species’ abundances; these effects are then compounded by sequencing strategy—16S rRNA amplicons are constrained by variable-region/primer bias and limited taxonomic resolution, whereas shotgun metagenomics resolves species and functional potential but at higher cost and with results contingent on database choice and depth—further complicating comparability across studies (31). Third, differences in sequence quality control, reference databases for taxonomic assignment (e.g., SILVA, Greengenes), and downstream statistical/multivariable adjustments mean that two teams analyzing the very same raw data can reach different conclusions about which taxa change significantly, creating apparent contradictions in the literature and weakening external reproducibility (32). These issues are compounded by substantial population heterogeneity in diet, geography, medication use, and genetics—confounders that are rarely fully controlled in small, cross-sectional “snapshot” studies with limited power for causal inference. These methodological inconsistencies are a principal reason for conflicting reports and the failure to identify a universal IBS microbiome signature, underscoring the urgent need for large-scale, longitudinal, function-focused, multi-omics investigations conducted under standardized, end-to-end protocols (sampling–extraction–sequencing–analysis) to yield more robust and reproducible findings.

Beyond taxonomy, functional readouts better align with IBS phenotypes: IBS-D often exhibits primary bile-acid perturbations and secretory/fast-transit features; IBS-C is frequently associated with methanogen enrichment and slow transit; IBS-M shows unstable/mixed profiles over time (**Table 3** for synthesized subtype-specific features and functional roles). Large-scale and longitudinal multi-omics studies are increasingly moving the field beyond taxonomy toward function. A multi-omics analysis integrating shotgun metagenomics, metabolomics, and host mucosal readouts reported an IBS signature with greater capacity to utilize fermentable carbohydrates, concordant with the benefit of restricting FODMAPs (33). Longitudinal multi-omics sampling further revealed subtype-specific pathways—e.g., higher unconjugated primary bile acids in IBS-D and altered purine metabolism with lower hypoxanthine—linking microbial functions to host epithelial and immune changes and to symptom flares (34). A 2024 cross-cohort metagenomic integration study (totaling 9,204 samples) was the first to identify a cross-geographically reproducible IBS microbial signature, discovering two enrichment patterns: one dominated by obligate anaerobes such as *Faecalitalea*, *Fusicatenibacter*, and *Ruminococcus*, and another rich in oral-like facultative anaerobes like *Streptococcus* and *Veillonella*. These patterns were associated with patient symptom severity, low-FODMAP diet, and rifaximin exposure (35). Population-scale analyses from the American Gut Project also demonstrate subtype-related functional differences (e.g., H_2_S production pathways in IBS-D and palmitoleate biosynthesis in IBS-C) and interactions with diet and mood symptoms (36). Machine-learning applications to metagenomes have delineated microbiota subtypes with therapeutic relevance and developed classifiers for IBS. Unsupervised stratification identified two IBS microbiota subtypes with distinct responses to the low-FODMAP diet (IBS^P *vs* IBS^H) (37). In addition, multi-class metagenomic models that include IBS have been trained on thousands of samples, supporting the feasibility of species-level feature sets for disease discrimination while underscoring the need for external validation and calibration across populations (38). Therefore, integrating multi-omics, longitudinal data, and machine learning is proving essential to move beyond taxonomic inconsistencies and uncover robust functional signatures that correlate with clinical phenotypes and treatment responses in IBS.

**Table 3:** Subtype-specific features and potential microbiome-targeted strategies.

IBS subtype	Microbiota/metabolite signals	Functional readouts	Candidate biomarkers	Potential strategies	Reference
IBS-D	↑ primary bile acids; Clostridia-rich signatures; reduced deconjugation	Faster transit; epithelial secretion; 5-HT signaling	Fecal primary BA↑; serum C4↑	Low-FODMAP; rifaximin; consider bile-acid sequestrants when BAM suspected; FMT	(201, 202, 205)
IBS-C	↑ methanogens (e.g., Methanobrevibacter smithii); methane-associated changes	Slower transit; gas dynamics	Breath methane↑	Low-FODMAP (selected responders); anti-methanogen strategies; synbiotics (strain-dependent)	(60, 204, 206)
IBS-M	Mixed/unstable profiles over time	Fluctuating motility and sensitivity	—	Personalized diet; brain–gut interventions	(36, 81)

IBS, irritable bowel syndrome; IBS-D: diarrhea-predominant irritable bowel syndrome; IBS-C, constipation-predominant irritable bowel syndrome; IBS-M, mixed-type irritable bowel syndrome; BA/BAs, bile acid/bile acids; C4, 7α-hydroxy-4-cholesten-3-one; 5-HT, 5-hydroxytryptamine; BAM, bile acid malabsorption; FMT, fecal microbiota transplantation; FODMAP, fermentable oligosaccharides, disaccharides, monosaccharides, and polyols; FODMAP, fermentable oligosaccharides, disaccharides, monosaccharides, and polyols; ↑/↓, increased/decreased.

## 3 Gut microbiota metabolites in IBS

### 3.1 SCFAs

Short-chain fatty acids (SCFAs) are primarily produced by gut microbiota through the anaerobic fermentation of carbohydrates. The main SCFAs include acetate, propionate, and butyrate. SCFAs serve as a key energy source, providing energy to colonic epithelial cells. Beyond providing energy, SCFAs are critical signaling molecules that modulate host immunity. Butyrate, for instance, is a potent histone deacetylase (HDAC) inhibitor in colonocytes and immune cells, leading to epigenetic changes that suppress inflammatory gene expression (39). Furthermore, SCFAs bind to G-protein-coupled receptors (GPCRs), such as FFAR2 (GPR43) and FFAR3 (GPR41), on the surface of both epithelial and immune cells. This activation can trigger downstream signaling that reinforces the gut barrier and promotes the differentiation of anti-inflammatory regulatory T cells (Tregs), thereby helping to maintain mucosal immune tolerance (40). Studies have shown that butyrate has protective effects on intestinal epithelial cells, promoting the expression of tight junction proteins, reducing intestinal permeability, and inhibiting the colonization of pathogenic microorganisms (39). Additionally, propionate and acetate participate in systemic energy metabolism and the regulation of inflammation (41). Some studies have reported a notable decrease in the fecal concentration of butyrate in IBS patients, which may lead to insufficient energy supply to colonic epithelial cells, impairing intestinal barrier function, increasing intestinal permeability, and exacerbating diarrhea and abdominal pain symptoms (42). Conversely, some IBS-D patients exhibit relatively higher levels of propionate and butyrate in their serum, suggesting that SCFA metabolism may have specific regulatory mechanisms that vary between IBS subtypes. A study by Gargari et al. recruited 240 non-constipated irritable bowel syndrome (NC-IBS) patients, including those with IBS-D and IBS-M, along with 100 healthy controls, to analyze fecal microbiota and SCFA levels (43). The results revealed significant differences in the fecal microbiota between NC-IBS patients and healthy controls, with healthy controls showing higher intra-individual biodiversity. Additionally, the non-constipated patients were classified into two subgroups based on their fecal SCFA levels (“high” and “low”), each with distinct bacterial characteristics. The “high” SCFA subgroup may represent a unique clinical phenotype of IBS, potentially offering insights for diagnosis and treatment. A recent double-blind randomized controlled trial (2025) conducted a 12-week probiotic intervention in patients with multiple IBS subtypes (44). The study found that from the 8th week onward, symptom severity in the treatment group was significantly lower than in the control group, accompanied by a significant increase in the levels of acetate, propionate, and butyrate. This increase in SCFAs was positively correlated with reduced intestinal permeability, upregulated expression of the tight junction proteins Occludin and Claudin-1, and a decrease in inflammatory markers. The researchers concluded that probiotics improve symptoms across all subtypes by increasing SCFA levels, repairing barrier function, and inhibiting inflammation, which further supports the “SCFA-barrier-clinical symptoms” pathway. SCFAs are consistently linked with the modulation of epithelial and immune pathways in IBS; however, effect directions and magnitudes vary across cohorts and subtypes, and causal inferences remain limited outside specific contexts (33). Current data support an associative—rather than uniformly causal—role for SCFAs that likely depends on host factors, transit, diet, and microbial context (45–47). In summary, while the relationship between SCFA levels and IBS symptoms is complex and varies by subtype and individual, their central role in modulating the intestinal barrier, immunity, and motility is well-established, positioning them as a key therapeutic target.

### 3.2 Bile acid metabolism

Bile acids (BAs) are primary bile acids synthesized in the liver from cholesterol through the catalysis of key enzymes such as cholesterol 7α-hydroxylase (CYP7A1), then excreted into the small intestine through the bile duct, where they primarily aid in the digestion and absorption of dietary lipids and fat-soluble vitamins (48, 49). In healthy individuals, gut microbiota modifies primary BAs into secondary BAs through specific enzymatic reactions, promoting their effective absorption in the ileum and facilitating their recycling via the enterohepatic circulation (50). However, this process is often disrupted in cases of dysbiosis. This microbial biotransformation is critical, as primary and secondary BAs have distinct and often opposing signaling properties. In a healthy gut, the pool of BAs is dominated by secondary BAs, which generally exert anti-inflammatory signals through receptors like the farnesoid X receptor (FXR). However, in IBS-D, dysbiosis often impairs the 7α-dehydroxylation step, leading to an accumulation of primary BAs in the colon (51). A study by Dior et al. found that in IBS-D patients, the levels of primary bile acids in feces (such as chenodeoxycholic acid, which promotes bowel movement) were significantly elevated, while bile acid deconjugation activity was reduced, indicating a weakened microbial ability to modify bile acids (52). In IBS-D patients, impaired bile acid malabsorption (BAM) correlates positively with accelerated colonic transit time, which is influenced by the composition of the gut microbiota (53). Additionally, gut microbiota alterations impact the efficiency of bile acid absorption in the ileum, reducing the activity of the apical sodium-dependent bile acid transporter (ASBT), leading to an increased flow of bile acids into the colon (54). A study by Zhao et al. indicates that IBS-D patients have elevated levels of total BAs and *Clostridia* (55). The study also found a positive correlation between bile acids in the stool and serum C4 (7-α-hydroxy-4-cholesten-3-one) with *Clostridia* levels. This suggests that a *Clostridia*-rich microbiota may promote bile acid synthesis and excretion in IBS-D patients by shortening gastrointestinal transit time and increasing stool water content. The abnormal accumulation of bile acids in the colon can exert multiple effects. First, their detergent-like properties can directly damage the epithelial barrier by disrupting tight junctions. Second, they stimulate colonic epithelial cells to secrete sodium and water, increasing the liquid content of the colon. Third, BAs are potent immune modulators; in experimental models, bile acids induce visceral hypersensitivity by activating a mucosal mast-cell–to-nociceptor pathway that operates through an FXR–NGF–TRPV1 axis, thereby driving immune activation and nociceptor sensitization (56). Furthermore, bile acids bind to the Takeda G-protein-coupled receptor 5 (TGR5) receptor on intestinal neurons, promoting the release of serotonin (5-HT), which regulates motility and sensitivity in the gut (57). These effects are closely linked to common IBS-D symptoms such as diarrhea, abdominal pain, and visceral hypersensitivity. Research on the bile acid-receptor axis has shown that in IBS-D patients, there is a decrease in *Bacteroides ovatus* while total and primary bile acids (such as chenodeoxycholic acid) are significantly increased (58). These bile acids activate the TGR5 receptor, leading to its upregulation in the small intestine and colon epithelium and inducing visceral hypersensitivity, an effect that can be reversed by TGR5 antagonists. Transplanting fecal matter from these patients into rats reproduced the mucosal barrier disruption and hyperalgesia, while a TGR5 inhibitor was able to ameliorate this phenotype. This confirms a causal link between the gut microbiota-bile acid-TGR5 axis and barrier function, as well as symptoms. Therefore, dysbiosis-driven alterations in bile acid metabolism, particularly through the TGR5 receptor signaling pathway, have emerged as a key mechanism explaining the symptoms of diarrhea and abdominal pain in IBS-D.

### 3.3 Gas metabolites

Gut microbiota fermentation also produces gas metabolites, which play an essential role in regulating gut physiological functions. Common gas metabolites include methane, hydrogen (H_2_), and hydrogen sulfide (H_2_S). These gases are not only by-products of microbial energy metabolism but also affect the host by altering intraluminal pressure, stimulating gut neurons, and modulating motility (59). Methanogens, such as *Methanobrevibacter smithii*, are major methane-producing archaea in the gut, while sulfate-reducing bacteria like *Desulfovibrio* spp. produce H_2_S (60). The generation and release of these gases constitute a dynamic process influenced by substrate availability, gut pH, and interactions between microbial groups. Numerous studies have shown a close relationship between gas metabolites and IBS symptoms. In IBS-C patients, methane production is typically high, and methane is believed to slow down gut motility and worsen constipation. In contrast, IBS-D patients often have excessive production of hydrogen and H_2_S, leading to symptoms like bloating, abdominal pain, and flatulence (60, 61). Excess gas accumulation alters intraluminal pressure, potentially stimulating intestinal nerve endings and triggering visceral hypersensitivity. The quantity of gas produced in the gut correlates positively with the severity of IBS symptoms, indicating that modulating gas production or promoting gas expulsion may help alleviate IBS symptoms.

Beyond their mechanical effects, these gases act as signaling molecules or “gasotransmitters” with distinct biological impacts. In sulfidogenic states, the overgrowth of sulfate-reducing *Desulfovibrio* spp. can trigger epithelial damage and the *in-vivo* release of pro-inflammatory cytokines, exacerbating experimental colitis and demonstrating the involvement of microbial H_2_S in mucosal immune activation and barrier disruption (62). In this context, H_2_ serves as the electron donor/substrate for sulfate-reducing bacteria like *Desulfovibrio* to produce H_2_S; in high-sulfide environments, H_2_ promotes H_2_S production via “substrate provision,” thereby exacerbating epithelial damage and pro-inflammatory responses (63). Furthermore, concentration-controlled H_2_S in a human gut-on-a-chip model also increased paracellular permeability and epithelial stress responses in a dose-dependent manner, providing a direct mechanistic link from sulfide excess to barrier breach and downstream immune activation. At the neuro-immune interface, sulfide and polysulfide donors trigger visceral pain-like behaviors via the TRPA1/Ca_v_3.2 pathway; the expression of TRPA1 on both gut afferent nerves and immune cells supports H_2_S-driven nociceptor sensitization and neuro-immune crosstalk relevant to visceral hypersensitivity (64). Regarding methane, intestinal methanogenic archaea (e.g., *Methanobrevibacter smithii*) can be recognized by human dendritic cells and induce the production of pro-inflammatory cytokines, indicating that archaeal components can directly trigger mucosal innate immunity. Moreover, the slow transit associated with methane production prolongs the contact time between bacterial products (such as LPS) and the epithelium, which can amplify PRR-mediated mucosal immune activation and low-grade inflammation (65). Current intervention strategies targeting gas metabolism, such as dietary changes, probiotic supplementation, or specific antimicrobial treatments to reduce gas-producing bacteria, may help relieve IBS-related symptoms. In conclusion, gas metabolites represent a direct physical link between microbial fermentation and cardinal IBS symptoms like bloating, pain, and altered bowel habits, making them a critical target for both diagnostic assessment (e.g., breath testing) and therapeutic intervention.

### 3.4 Tryptophan metabolites

Tryptophan is an essential amino acid that is not only a building block for protein synthesis but also serves as a precursor for neurotransmitters and other bioactive substances, such as serotonin and melatonin, which have a significant impact on the gut-brain axis (66). Tryptophan is primarily metabolized in the host via the kynurenine pathway and the serotonin pathway (67). The generation of serotonin (5-HT) relies on the catalytic action of tryptophan hydroxylase (TPH), which is closely involved in regulating gut motility, secretion, visceral hypersensitivity, abdominal pain, and neuroregulation (68). Studies have shown that in IBS-D patients, the level of 5-HT in the colon is significantly elevated, possibly due to an imbalance in the tryptophan metabolism pathway (69). Additionally, gut microbiota can directly convert tryptophan into various indoles and their derivatives. Many of these indoles, such as indole-3-propionic acid (IPA) and indole-3-aldehyde, are potent ligands for the aryl hydrocarbon receptor (AhR), a transcription factor expressed on intestinal epithelial cells and many immune cells, including innate lymphoid cells (ILCs) and T cells (70, 71). AhR activation is a cornerstone of mucosal immunity. When activated by microbial indoles, it stimulates ILCs and T helper 17 (Th17) cells to produce interleukin-22 (IL-22), a key cytokine that reinforces epithelial barrier function by promoting epithelial cell proliferation and inducing the expression of antimicrobial peptides (72, 73). Furthermore, AhR signaling helps maintain immune tolerance by promoting the development of Tregs (74). In IBS, several studies have reported reduced levels of fecal indole derivatives and evidence of impaired AhR activation in the mucosa, particularly in IBS-D. This deficiency can lead to decreased IL-22 production, a compromised epithelial barrier, and a pro-inflammatory shift in mucosal immune tone (75). A lack of AhR agonists may lead to a reduction in glucagon-like peptide-1 (GLP-1) and IL-22 secretion, thereby increasing intestinal permeability and exacerbating inflammation, which further worsens IBS symptoms (76). Some studies suggest that the ability of microbiota to convert tryptophan into AhR agonists diminished, which may be closely related to metabolic disorders and symptom exacerbation in IBS patients (77). Therefore, regulating the balance of tryptophan and its metabolites not only helps improve gut function but may also have positive effects on IBS-related neuropsychiatric symptoms. However, a 2025 Mendelian randomization study revealed that genetically predicted IBS is associated with elevated plasma levels of tryptophan, serotonin, and kynurenine, whereas genetically predicted levels of tryptophan metabolites have no significant impact on IBS risk (78). This result implies that IBS may in turn drive disturbances in tryptophan metabolism, contributing to a vicious cycle of neuro-immune dysregulation. Overall, the tryptophan metabolic pathway represents a critical node where the gut microbiota influences neuroendocrine and immune regulation; its bidirectional dysregulation is implicated not only in gastrointestinal symptoms but may also help explain the high comorbidity between IBS and mood disorders.

## 4 Gut microbiota and the pathological mechanisms of IBS

The gut microbiota is closely implicated in the pathophysiology of IBS. It influences the clinical symptoms of IBS patients by regulating the epithelial barrier, immune response, gut-brain axis, and visceral sensation. This section will explore these key processes and their interactions, providing new insights into the underlying pathology of IBS.

### 4.1 Gut barrier function and permeability

The intestinal epithelium forms the largest interface between the body and the external environment, and their integrity is crucial for maintaining host immune homeostasis, nutrient absorption, and defending against the invasion of external pathogens. Under normal physiological conditions, commensal bacteria such as *Bifidobacterium*, *Lactobacillus*, and *Faecalibacterium prausnitzii* colonize the gut. They ferment dietary carbohydrates to produce short-chain fatty acids (SCFAs), which provide essential energy for colonocytes and upregulate the expression of tight junction proteins (such as ZO-1, claudin, and occludin), thereby enhancing cell adhesion and maintaining the structural integrity of the epithelial barrier (79). However, studies have shown that in IBS patients, especially in the IBS-D subtype, the abundance of beneficial bacteria producing butyrate significantly decreases, accompanied by abnormal changes in SCFA composition and concentration. This leads to insufficient energy supply for epithelial cells, reduced expression of tight junction proteins, and a weakened gut mucosal barrier (43). Once the barrier is damaged, bacteria, toxins (such as LPS), and other antigens are more easily able to penetrate the epithelial layer, enter the submucosa, and even the bloodstream, triggering local and systemic inflammatory responses. This phenomenon is known as “leaky gut” (80). Clinically, many IBS patients show signs of increased intestinal permeability, elevated levels of pro-inflammatory cytokines in the serum, and enhanced immune activation (81). Further research has pointed out that dysbiosis not only reduces SCFA production but is also associated with an increase in bacteria that degrade the mucus layer (such as *Ruminococcus gnavus* and *Ruminococcus torques*), which secrete mucin-degrading enzymes and impair the mucus layer covering the epithelial surface, further weakening the physical barrier function (82). In addition to the lack of mucus and SCFAs caused by dysbiosis, a 2025 probiotic randomized controlled trial has also shown that by increasing SCFA levels and reducing intestinal permeability, the expression of tight junction proteins such as Occludin, Claudin-1, and Zonulin significantly improved between weeks 8 and 12 (44). Symptom improvement was positively correlated with the increase in SCFAs (r = 0.43, *P* = 0.002), further demonstrating the importance of restoring barrier function in IBS treatment. Studies have also found that supplementation with probiotics such as *Lactobacillus rhamnosus* can repair epithelial barrier function by inducing tight junction protein expression and increasing mucus secretion (83). This finding offers a new therapeutic approach for improving the intestinal barrier integrity in IBS patients through microbiome intervention. In essence, a compromised intestinal barrier, or “leaky gut,” driven by microbial dysbiosis and reduced SCFA production, is a central pathophysiological mechanism that translates microbial shifts into the low-grade inflammation and immune activation characteristic of IBS.

### 4.2 Immune system regulation

The gut immune system is an essential component in maintaining the balance between the body and the external environment, and its normal function relies on colonization by the gut microbiota and the regulation of their metabolic products. Under normal conditions, commensal microbiota interact with gut epithelial cells, dendritic cells, mast cells, macrophages, and other immune cells by secreting SCFAs, indolic compounds, and other signaling molecules, maintaining local immune tolerance and an anti-inflammatory balance (11). However, in IBS patients, many studies have shown that dysbiosis is closely related to local low-grade inflammation and immune activation (84). Pro-inflammatory cytokines such as TNF-α, IL-1β, IL-6, and IL-8 are often elevated in the intestinal mucosa of IBS patients, while anti-inflammatory cytokine IL-10 is relatively decreased (85). This inflammatory state may partially stem from stress-induced activation of the HPA axis and stimulation of the immune system by bacterial cell wall components (such as LPS). In IBS patients, the number of immune cells in the gut lamina propria, such as mast cells, T cells, lymphocytes, and macrophages, significantly increases (86). Mast cells, due to their proximity to nerve endings, serve as key mediators of visceral hypersensitivity (VH) (87). A recent study has shown that in the mucosal supernatant of IBS patients, elevated levels of histamine, serotonin, and serine proteases (such as trypsin-3 and tryptase) released from mast cells significantly enhance the excitability of colorectal sensory nerves (85). Blocking the histamine H1 receptor or protease activity can reverse this neural hyperexcitability. These mediators drive visceral hypersensitivity by promoting the phosphorylation and sensitization of pain receptors like TRPV1/4 and TRPA1 via the phosphatidylinositol signaling pathway and protease-activated receptor 2 (PAR2). This reveals the therapeutic potential of targeting mast cells and pain-related ion channels.

In addition to local inflammation, post-infectious IBS (PI-IBS) is also thought to be related to long-term immune activation (88). Mucosal damage caused by pathogens, the loss of interstitial cells of Cajal, and functional changes in enterochromaffin cells can lead to persistent immune activation and increased visceral sensation (89). Some studies show that in PI-IBS patients, the number of T cells and mast cells in the mucosa increases, with immunohistochemistry revealing elevated levels of pro-inflammatory cytokines such as IL-4, IL-1β, and TNF-α, while anti-inflammatory cytokine levels are decreased (90). These changes collectively disrupt the intestinal barrier and immune tolerance, forming a vicious cycle. Additionally, bacterial components in the gut, such as flagellin and LPS, can act as ligands for Toll-like receptors (TLRs) (91). The gut immune system can recognize and respond to changes in the microbiota through pattern recognition receptors (PRRs) such as TLRs. TLR4 and TLR5 expression is upregulated in IBS patients, further activating pro-inflammatory cascades (92). Overall, a complex regulatory network exists between the gut microbiota and the immune system, determining local immune tolerance and anti-inflammatory states, while also triggering inflammatory responses during dysbiosis, leading to visceral hypersensitivity and other IBS symptoms. Thus, the dysbiotic microbiota in IBS disrupts immune homeostasis, shifting the balance from tolerance towards a state of chronic, low-grade mucosal inflammation and immune activation, which directly contributes to symptom generation, particularly visceral pain.

### 4.3 Gut-brain axis

The gut-brain axis is a bidirectional communication network composed of the central nervous system, autonomic nervous system, enteric nervous system, endocrine system, and gut microbiota. Its dysfunction is closely related to visceral hypersensitivity, gastrointestinal motility abnormalities, and mood disorders in IBS patients. The gut microbiota and its metabolites are now recognized as critical regulators of this axis, influencing brain function and behavior through at least three interconnected pathways: neural, endocrine, and immune (8). Neuroimaging studies in IBS patients have revealed structural and functional changes in key brain areas, and recent work has begun to link these neural signatures to specific microbial profiles. For example, a study by Labus et al. found that the functional connectivity between brain regions such as the thalamus, basal ganglia, and prefrontal cortex was significantly correlated with the abundance of genera like Fusobacterium and Bacteroides, providing human evidence for a “microbe-neurocircuit” coupling (93).

The primary and most rapid of these pathways is the neural (vago-enteric) route, a direct line from the gut lumen to the brainstem. Microbial metabolites can directly or indirectly engage sensory pathways that ascend via the vagus nerve to brainstem nuclei controlling pain, arousal, and stress (94). Enterochromaffin (EC) and other enteroendocrine cells (EECs) sense luminal cues and microbial products, releasing serotonin (5-HT) and other mediators that activate vagal afferents and local enteric neurons, thereby shaping visceral sensation (95, 96). Recent work has shown that bacterial tryptophan metabolites can induce 5-HT secretion via TRPA1^+^ enteroendocrine cells, thus modulating upstream sensory pathways. In pathological states, this pathway may amplify pain inputs and promote visceral hypersensitivity (95). The endocrine (neuroendocrine) pathway serves as a crucial bridge, where the gut microbiota acts as a key regulator of EEC function. For instance, the gut microbiota can modulate L-cells to secrete glucagon-like peptide-1 (GLP-1) and peptide YY (PYY) (97). Once in circulation, these hormones not only regulate local gut function but also influence central appetite, stress, and mood-regulating networks by acting on the hypothalamus or via vagal pathways (98, 99). Furthermore, microbial metabolites like SCFAs can indirectly activate the vagus nerve by stimulating EECs to release multiple hormones, such as 5-HT and GLP-1. This vagal activation transmits signals from the gut to the brainstem, influencing downstream neural circuits involved in mood, stress responses, and the perception of visceral pain (100). Under the dysbiotic conditions of IBS (e.g., a reduction in SCFA-producing bacteria), the secretion patterns of these hormones can be altered, leading to abnormal gut motility and disordered sensory signaling, which in turn impacts brain function. The immune (neuro-immune) pathway describes the central effects of barrier disruption and inflammatory signaling. Dysbiosis and impaired barrier function increase the translocation of microbe-associated molecular patterns, such as LPS, which can induce a systemic inflammatory state and the release of cytokines such as IL-6 and TNF-α (101). These cytokines can enter the brain through active transport or a compromised blood-brain barrier, or they can influence brainstem nuclei via vagal afferents, inducing “sickness behavior,” anxiety, and altered pain processing (102). More directly, SCFAs can cross the blood-brain barrier and act on microglia. Recent *in vivo*, *in vitro*, and review evidence shows that SCFAs like propionate and butyrate can inhibit microglial HDAC activity and the NF-κB pathway, shaping an anti-inflammatory and neurotrophic phenotype, thereby altering the reactivity of circuits related to pain and mood (103, 104). This “microbe-immune-brain” crosstalk provides a biological pathway to explain the high comorbidity between IBS and disorders like anxiety and depression.

Moreover, the gut microbiota itself is a veritable factory of neuroactive molecules, capable of directly synthesizing or modulating various neurotransmitters crucial to the gut-brain axis. For instance, many beneficial strains, particularly within the genera *Lactobacillus* and *Bifidobacterium*, are known to produce the primary inhibitory neurotransmitter, gamma-aminobutyric acid (GABA) (105). Locally in the gut, GABA can modulate the activity of the enteric nervous system (ENS), thereby influencing intestinal motility and dampening visceral pain signals (106). In IBS, a reduction in GABA-producing bacteria may lead to a weakening of this inhibitory tone, thus contributing to visceral hypersensitivity and anxiety. Similarly, certain strains, such as *Bacillus*, can synthesize catecholamines, including dopamine and norepinephrine (105). While these peripherally produced macromolecules do not readily cross the blood-brain barrier, they can locally regulate motility, secretion, and immune cell function within the gut and transmit signals to the brain via the vagus nerve, affecting mood and stress responses (107). The aforementioned indolic compounds, particularly tryptamine, serve as a prime example of how a microbial metabolite can directly “hijack” and amplify host neural signaling, as its structural similarity to serotonin allows it to stimulate 5-HT release from enterochromaffin cells (108). Therefore, in the dysbiotic state of IBS, the composition of this “neurotransmitter soup” becomes imbalanced. This dysregulation not only disrupts local gut physiology but also sends an aberrant flow of signals to the central nervous system, thereby contributing to both the core symptoms of IBS (pain, altered bowel habits) and its common psychological comorbidities. In summary, the gut-brain axis is the critical bidirectional highway where these interconnected neural, endocrine, and immune pathways converge. Microbial dysbiosis can initiate or perpetuate dysfunction along this axis, ultimately translating gut-level disturbances into the central nervous system changes that define IBS as a disorder of gut-brain interaction.

### 4.4 Visceral hypersensitivity

Visceral hypersensitivity (VH) is one of the most prominent features of IBS pathophysiology, characterized by abnormal, intense pain or discomfort in response to normal, harmless physiological stimuli (109). The composition and dysfunction of the gut microbiota play a crucial role in the occurrence and development of visceral hypersensitivity in IBS patients. Proper bacterial colonization after birth affects pain pathways, with germ-free mice initially exhibiting blunted responses to inflammatory pain (110). Furthermore, antibiotic-induced visceral hypersensitivity models further confirm the key role of gut microbiota in regulating visceral pain, with this effect closely related to the duration of antibiotic exposure. Mice exposed to antibiotics early in life develop visceral hypersensitivity as adults, while antibiotic treatment in adulthood can reduce visceral pain responses induced by intraperitoneal acetic acid or colonic injections of capsaicin, while paradoxically increasing sensitivity to colorectal distension (CRD) stimuli (111). Recent studies using a germ-free (GF) mouse model with fecal microbiota transplantation have shown that transplanting microbiota from IBS patients induces pronounced visceral hypersensitivity in the mice, while transplanting microbiota from healthy controls maintains normal pain thresholds (112). Some probiotics, such as *Lactobacillus reuteri*, have been shown to partially reverse visceral hypersensitivity by regulating the expression of pain receptors like TRPV1 and reducing the release of local inflammatory mediators (113). In clinical research, Symprove (a multi-strain probiotic containing *Lactobacillus rhamnosus*, *Lactobacillus plantarum*, *Lactobacillus acidophilus*, and *Bifidobacterium breve*) has been shown to significantly improve overall symptom severity in IBS patients (114). Additionally, *Bifidobacterium* MIMBb75 has been shown to significantly improve symptoms such as abdominal pain, bloating, urgency, and digestive disturbances, thereby enhancing the quality of life of patients (115). This further supports the key role of dysbiosis in the development of visceral hypersensitivity and suggests that regulating the gut microbiota may be a new strategy for treating IBS-related visceral pain. Ultimately, visceral hypersensitivity stands as a core symptom generator in IBS, where microbial dysbiosis, immune mediators, and altered gut-brain signaling converge to lower the pain threshold, transforming normal physiological events into painful experiences.

### 4.5 Stress

In recent years, extensive research has confirmed that stress plays a critical important role in the pathogenesis of IBS, not only by directly affecting the activation and regulation of the hypothalamic-pituitary-adrenal (HPA) axis but also by altering the gut microbiota, disrupting the epithelial barrier, and activating local immune responses, further exacerbating visceral hypersensitivity and triggering or worsening IBS symptoms (116). Chronic psychological stress or acute stress can significantly alter the diversity and composition of the gut microbiota (117). Studies have found that prenatal and postnatal stress affect the initial colonization and long-term stability of the microbiota, potentially causing persistent neurodevelopmental and immune dysregulation, providing a foundation for IBS development later in life (118). Further studies suggest that early stress models, such as maternal separation, cause anxiety and visceral hypersensitivity in adult mice, a phenomenon that is less pronounced in germ-free animals, further proving the crucial mediating role of microbiota in stress-induced IBS (119). Clinical studies have found that IBS patients often exhibit abnormal cortisol secretion, with plasma cortisol levels and responses to adrenocorticotropic hormone (ACTH) differing from those of healthy individuals, suggesting that long-term stress may promote IBS onset through abnormal activation of the HPA axis (86). Additionally, chronic stress leads to sustained sympathetic nervous system activation, raising levels of pro-inflammatory cytokines (such as IL-6, IL-8, and TNF-α), further triggering local inflammatory responses and impairing gut epithelial barrier function (120). The systemic and local inflammation induced by stress provides a permissive environment for gut microbiota dysbiosis, which in turn worsens intestinal inflammation and disrupts barrier function, creating a vicious cycle. In conclusion, stress acts as both a trigger and an amplifier in IBS pathophysiology, directly impacting the gut-brain axis while also shaping a pro-inflammatory gut environment that fosters dysbiosis, thereby locking the system in a self-perpetuating cycle of symptoms.

## 5 Microbiome-based treatment strategies for IBS

**Figure 2:**
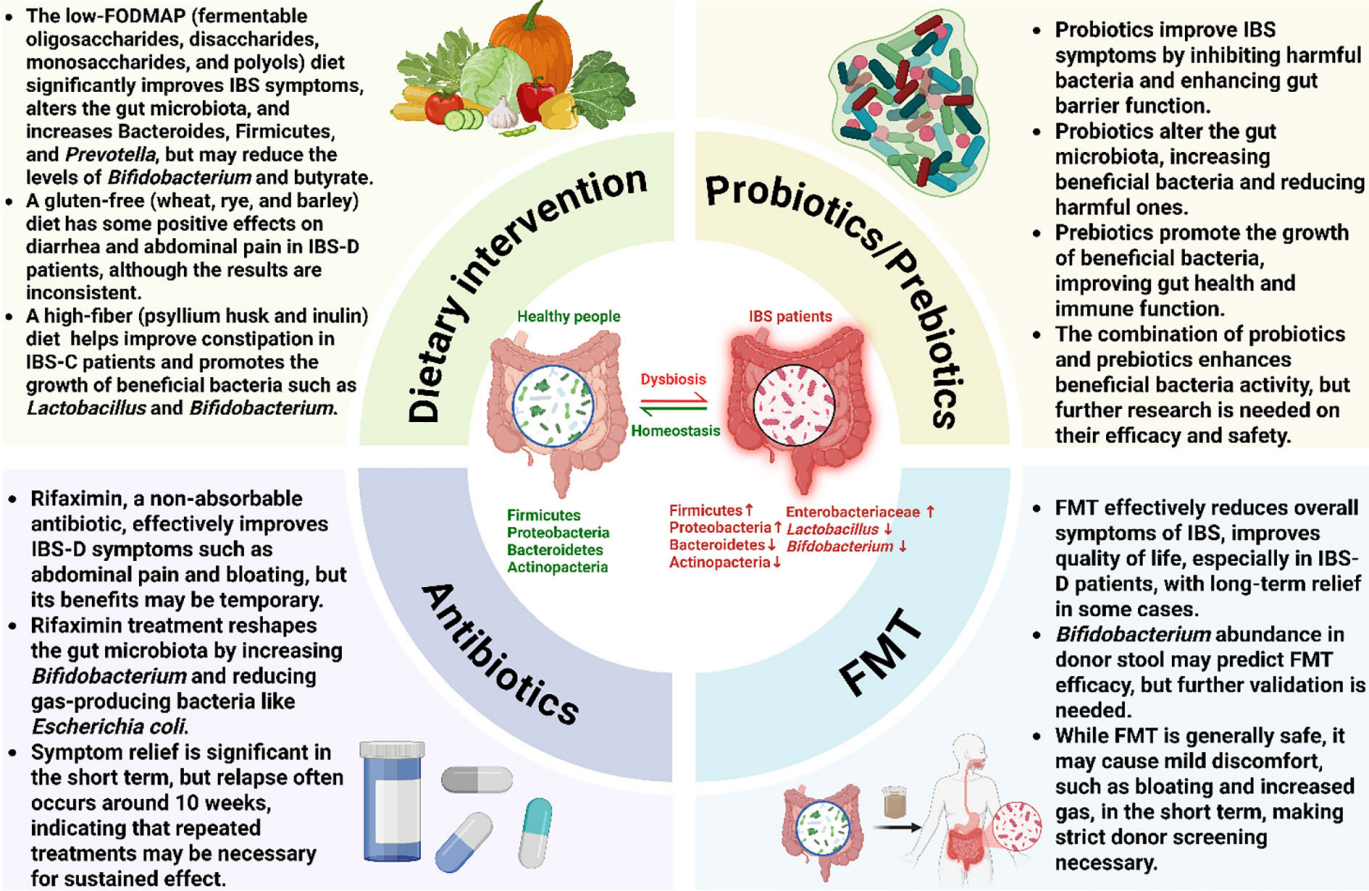
Microbiome-targeted interventions for irritable bowel syndrome. This figure provides a comprehensive overview of therapeutic strategies aimed at modulating the gut microbiota to manage IBS. The central panel illustrates the transition from a healthy microbial homeostasis to the dysbiotic state often seen in IBS, which is characterized by an altered bacterial composition, including an increase in Firmicutes, Enterobacteriaceae and Proteobacteria and a decrease in Bacteroidetes, Actinobacteria, *Lactobacillus*, and *Bifidobacterium*. The four surrounding quadrants detail the primary interventions: Dietary interventions (e.g., low-FODMAP, high-fiber) modify microbial composition and function by altering nutrient availability; Probiotics and prebiotics restore balance by introducing or promoting beneficial bacteria to improve gut barrier function; Antibiotics (Rifaximin) reduce specific pathogenic or gas-producing bacteria; and fecal microbiota transplantation (FMT) aims to comprehensively reset the gut ecosystem by introducing a healthy donor microbiota. Ultimately, each of these strategies seeks to correct dysbiosis and restore microbial balance to alleviate the symptoms of IBS.

Microbiome-based treatments have gained significant attention in the management of IBS. The primary goal of these therapies is to counteract the gut dysbiosis commonly observed in patients, which is often characterized by an altered bacterial composition, such as an increase in Firmicutes, Enterobacteriaceae and Proteobacteria, and a decrease in beneficial groups like *Lactobacillus*, and *Bifidobacterium*. As illustrated in **Figure 2**, these strategies include dietary interventions that modify nutrient availability, probiotics and prebiotics to introduce or promote beneficial bacteria, antibiotics like rifaximin to reduce specific pathogenic or gas-producing bacteria, and FMT to comprehensively reset the gut ecosystem. Each of these approaches aims to modify the gut microbiome to restore balance and improve overall gut health, thereby alleviating symptoms. In **Table 4**, we summarize therapeutic approaches for IBS based on the microbiome over the last decade. An overview of these interventions and their evidence tier is provided in **Table 5**.

**Table 4:** Summary of microbiota-targeted treatments for IBS.

Treatment method	Model (clinical/preclinical)	Study design/sample size	Administration route	Dosing regimen (dose & duration)	Main findings	Reference
Probiotic (Lacidophilin tablet)	Preclinical (rat IBS model)	Controlled experiment in IBS model rats (n=8/group)	Oral gavage	0.84 g/kg/day, 2 weeks	Reduced visceral hypersensitivity and abnormal motility; alleviated anxiety/depressive behavior; restored mucus barrier proteins; modulated gut microbiota; reduced gut inflammation.	Fan et al., 2025 (207)
Probiotic (B. longum NCC3001)	Clinical (IBS patients)	Pilot RCT, n=44	Oral capsules	(1.0E + 10 colony-forming units/1 g powder with maltodextrin) Daily, 10 weeks	No significant improvement in IBS symptoms or anxiety at 6 weeks; QoL improved; by 10 weeks, lower depression scores than placebo.	Pinto-Sánchez et al., 2017 (208)
Probiotics (multi-strain)	Clinical (IBS-D adults)	Systematic review & meta-analysis (10 RCTs, n=943)	Oral (various formulations)	Varied strains; most <12 weeks	Reduced global IBS-D symptoms, abdominal pain, and bloating *vs* placebo; no significant QoL difference.	Wang et al., 2022 (209)
Probiotics (mixed strains)	Clinical (IBS-C adults)	Systematic review & meta-analysis (10 RCTs, n=757)	Oral (various formulations)	4–8 weeks	Improved stool consistency and increased fecal *Bifidobacterium*/*Lactobacillus*; no significant improvement in pain, bloating, or IBS-QoL.	Shang et al., 2022 (210)
Probiotics (various strains)	Clinical (IBS patients)	Systematic review & meta-analysis (82 RCTs, n=10,332)	Oral (various)	4–12 weeks	Moderate-certainty: some single strains (e.g., specific *E. coli*) improve overall IBS; lower-certainty for some *Lactobacillus* (e.g., *L. plantarum* 299V) and combinations; low-certainty modest pain relief with specific yeasts or *Bifidobacterium* strains.	Goodoory et al., 2023 (137)
Prebiotics (FOS, inulin, etc.)	Clinical (IBS patients)	Systematic review & meta-analysis (11 RCTs, n=729)	Oral supplements	≤6 g/day *vs* higher; 4–12 weeks	No overall difference *vs* placebo in pain, bloating, flatulence, or QoL. Low doses (≤6 g/day) and non-inulin FOS improved bloating; higher inulin-type FOS worsened bloating. Increased fecal *Bifidobacterium*.	Wilson et al., 2019 (211)
Prebiotic (inulin-type fructan)	Clinical (IBS-C patients)	Randomized crossover trial, n=47	Oral (inulin *vs* control)	5000 mg of inulin: one packet daily for the first week, followed by two packets daily for the next three weeks. After 28 days, the two groups switch.	After inulin: pain was reduced by approximately 68%, and bloating was reduced by approximately 35%; stool frequency/consistency improved. No significant differences *vs* control condition overall.	Bărboi et al., 2022 (212)
Prebiotic (short-chain FOS)	Clinical (IBS patients)	Double-blind RCT, n=79	Oral (scFOS *vs* placebo)	5 g/day, 4 weeks	Fecal *Bifidobacterium* increased in scFOS group; no significant between-group difference for *Bifidobacterium* change; most other bacteria unchanged.	Azpiroz et al., 2016 (213)
Antibiotic (rifaximin)	Clinical (IBS-D patients)	Double-blind RCT (TARGET trials), n=1,074	Oral 550 mg tablet	2 weeks TID; repeat for relapses	c	Lembo et al., 2016 (149)
Antibiotic (rifaximin)	Clinical (IBS-D patients)	RCT, n=103	Oral 550 mg tablet	2 weeks TID; repeat for relapses	Short-term decreases in 7 taxa (e.g., *Streptococcus*, *Microbacterium*, Enterobacteriaceae) after 2 weeks; changes transient—none persisted by week 46.	Fodor et al., 2019 (148)
Antibiotic (rifaximin)	Clinical (IBS patients)	Systematic review & meta-analysis (5 RCTs, n=1,800)	Oral 400–550 mg tablet	2 weeks TID per course	No significant benefit *vs* placebo for global relief or abdominal pain; consistently greater bloating relief *vs* placebo.	Black et al., 2020 (214)
Antibiotic (rifaximin) + Probiotic	Clinical (IBS patients)	RCT, n=70	Oral (tablets & capsules)	Rifaximin (200 mg, four times daily for 14 days) and probiotics (1×10^10^ CFU, once daily for 28 days), evaluated over 8 weeks	Combination therapy achieved symptom relief rates of 65.7% at weeks 4 and 8, *vs* 31.4% with rifaximin alone; quality−of−life improvement was higher in the combination group (65.7% *vs* 37%).	Oh et al., 2025 (150)
FMT (donor stool *vs* placebo)	Clinical (moderate–severe IBS)	Double-blind RCT, n=83	Colonoscopic infusion (donor *vs* autologous)	Single infusion (50–80 g of faeces)	3 mo: adequate relief 65% (FMT) *vs* 43% (placebo); lower IBS-SSS in FMT; 12 mo sustained response 56% *vs* 36%.	Johnsen et al., 2018 (158)
FMT (30 g *vs* 60 g *vs* placebo)	Clinical (IBS patients)	Double-blind RCT, n=165	Colonoscopic infusion	Single 30 g or 60 g dose	Dose-responsive efficacy: responders 23.6% (placebo) *vs* 76.9% (30 g) and 89.1% (60 g); microbiota shifts correlated with symptom improvement.	El-Salhy et al., 2020 (156)
FMT (capsule *vs* enema *vs* placebo)	Clinical (IBS patients)	Double-blind RCT, n=45	Oral capsules *vs* rectal enema *vs* placebo	Single 50 g dose	Significant symptom improvement with both capsule and enema; response 86.7% (capsule) and 73.3% (enema) *vs* 26.7% (placebo).	Aumpan et al., 2025 (160)
FMT (meta-analysis)	Clinical (IBS patients)	Systematic review & meta-analysis (9 RCTs, n=516)	Various (mostly colonoscopic/oral)	The fecal FMT group received a fecal dose of 30–80 g, while the capsule FMT group received a fresh fecal dose of 14.25–600 g (50 g/day × 12 days)	Single FMT reduced IBS-SSS at 1, 3, 6, 24, 36 months; higher remission rates and improved IBS-QoL at 3, 24, 36 months; no increase in serious AEs.	Wang et al., 2023 (157)
Diet: fiber supplementation	Clinical (IBS-C patients)	Systematic review (3 RCTs, n=381)	Oral (psyllium, bran, etc.)	4–12 weeks	Beneficial across trials in IBS-C, improving stool frequency/consistency (psyllium most consistent; bran mixed in other literature).	Rao et al., 2015 (215)
Diet: low-FODMAP *vs* regular	Clinical (IBS patients)	Meta-analysis (10 studies, n=550)	Dietary instruction	4–13 weeks; IBS-SSS outcome	Both improved symptoms, but low-FODMAP led to greater IBS-SSS reduction; significantly lower post-diet IBS-SSS (p=0.002).	Varjú et al., 2017 (167)
Diet: low- *vs* high-FODMAP	Clinical (IBS patients)	Randomized crossover trial, n=37	Dietary intervention	3 weeks	Low-FODMAP increased Actinobacteria (esp. *Bifidobacteria*) and Firmicutes (*Clostridiales*) *vs* high-FODMAP; no significant α/β-diversity change *vs* baseline.	McIntosh et al., 2017 (216)
Diet: low-FODMAP (microbiome effects)	Clinical (IBS patients)	Systematic review & meta-analysis (9 RCTs, n=403)	Dietary intervention	2–8 weeks	Low-FODMAP consistently reduced fecal *Bifidobacterium vs* controls; no consistent change in overall diversity or other major taxa; SCFAs similar to controls.	So et al., 2022 (217)
Diet: low-FODMAP elimination & reintroduction	Clinical (IBS patients)	Double-blind RCT (blinded reintroduction), n=117	Dietary intervention	The FODMAP and control powders, labeled A through G, were administered three times daily for seven consecutive days according to a randomized, blinded, crossover sequence	After 2 weeks, IBS-SSS improved markedly (80% responders). During blinded reintroduction, 85% relapsed; median 2–3 specific FODMAP triggers (most common: fructans 56%, mannitol 54%).	Van den Houte et al., 2024 (165)
Gluten-free diet *vs* Traditional Dietary Advice (TDA) *vs* Low-FODMAP diet (LFD)	Clinical (IBS, non-constipated)	3-arm RCT, n=99 (33 per arm), 4 weeks	Dietitian-guided diet	GFD: strict gluten avoidance; comparator arms received standardized TDA or LFD for 4 weeks	All three diets reduced IBS-SSS; responder rates similar (GFD 58%, LFD 55%, TDA 42). TDA most acceptable; no clear superiority of GFD over LFD.	Rej et al., 2022 (218)
GFD run-in then gluten-containing bread *vs* gluten-free bread (challenge)	Clinical (IBS)	Double-blind randomized placebo-controlled, n=60; 4-week GFD run-in then 4-week challenge	Diet (bread)	Two slices/day gluten-containing bread *vs* gluten-free bread for 4 weeks after GFD run-in	Symptoms improved on GFD; gluten bread significantly exacerbated IBS symptoms *vs* gluten-free bread during challenge.	Zanwar et al., 2016 (219)
Psyllium (ispaghula)	Clinical (pediatric IBS)	Double-blind RCT; n=81 (43 psyllium, 38 placebo)	Oral	Approximately 6–12 g per day (age-adjusted), 4 weeks	Reduced IBS-SSS versus placebo; 43.9% remission at 4 weeks; short-term benefit	Menon et al., 2023 (220)
Psyllium co-administered with inulin	Clinical (IBS and healthy volunteers)	Randomized, single-blind, crossover; n=36 IBS, 19 healthy	Oral (test drinks)	Inulin 20 g with or without psyllium 20 g	Psyllium attenuated inulin-induced colonic gas and symptoms in IBS	Gunn et al., 2022 (221)
Diet (low-FODMAP) + Probiotic	Clinical (IBS patients)	Double-blind RCT, n=85	Oral (diet & capsules)	Probiotic (10^9^ CFU each of B. lactis B420 & *L. acidophilus* NCFM) daily for 3 weeks	Both groups had >85% reduction in IBS−SSS; improvements in visual analogue scale (VAS) pain scores. Probiotic group showed better stool normalization—70.6% *vs* 35.3% (IBS−C) and 75.0% *vs* 58.8% (IBS−D)	Turan et al., 2021 (174)

RCT, randomized controlled trial; RCTs, randomized controlled trials; IBS, irritable bowel syndrome; IBS-D, diarrhea-predominant irritable bowel syndrome; IBS-C, constipation-predominant irritable bowel syndrome; IBS-SSS, irritable bowel syndrome symptom severity score; IBS-QoL, irritable bowel syndrome–quality of life; QoL, quality of life; FMT, fecal microbiota transplantation; FODMAP, fermentable oligo-, di-, mono-saccharides and polyols; GFD, gluten-free diet; LFD, low-FODMAP diet; TDA, traditional dietary advice; FOS, fructo-oligosaccharides; scFOS, short-chain fructo-oligosaccharides; TID, three times daily; AE, adverse event.

**Table 5:** Evidence map of microbiome-related interventions in IBS.

Intervention	Primary target	Best-supported subtype	Highest evidence tier
Low-FODMAP diet	Reduce fermentable substrates (FODMAPs)	IBS-D/IBS-M	★★★★ Meta-analyses of RCTs
Probiotics (strain-specific)	Barrier/immune modulation; gas dynamics	Mixed/strain-dependent	★★★ RCTs/meta (low–very low certainty)
Prebiotics/Synbiotics	Nurture beneficial taxa/↑SCFAs	Selected IBS-C; mixed overall	★★ Mixed RCTs
Rifaximin	Microbiota modulation (non-absorbed antibiotic)	IBS-D	★★★ Multiple RCTs/meta
FMT	Community reconstitution	Unclear (heterogeneous)	★★ Meta + neutral/negative RCTs in rigorous settings
Postbiotics	Defined bioactives (no live cells)	Exploratory	★ Mechanistic/small human studies
Engineered/gene-edited & synbio consortia	Programmable functions; designed consortia	Exploratory	★ Preclinical/phase 1

Stars denote the highest tier of supporting evidence—★ mechanistic/early-phase; ★★ single/small RCTs; ★★★ multiple RCTs/meta-analyses; ★★★★ meta-analyses of RCTs.

### 5.1 Probiotics and prebiotics

The Food and Agriculture Organization (FAO) and the World Health Organization (WHO) define probiotics as “live microorganisms which, when administered in adequate amounts, confer a health benefit on the host” (121). In terms of regulating gut microbiota, probiotics act to competitively inhibit pathogenic bacteria. Their mechanisms include: (1) directly inhibiting or killing pathogenic bacteria by producing bacteriocins, SCFAs, and biosurfactants (122); (2) competitively blocking pathogen adhesion to intestinal epithelial cells through specific adhesion proteins, thus reducing pathogen colonization in the gut (123); and (3) by lowering the local pH (e.g., producing SCFAs such as lactic acid, acetic acid, butyrate, and propionate), probiotics make the gut environment more acidic, inhibiting the growth of harmful bacteria that prefer neutral or alkaline environments (124). Several studies have shown that after 4 to 8 weeks of probiotic treatment, IBS patients experienced significant improvement in symptoms such as abdominal pain, bloating, and discomfort, with some patients also showing a normalization of bowel frequency and stool characteristics (125–127). A meta-analysis of 35 randomized controlled trials involving 3,452 patients with irritable bowel syndrome showed that, compared with placebo, patients taking probiotics had a lower rate of symptom persistence (RR 0.79, 95% CI 0.70–0.89, *P* < 0.0001). Furthermore, probiotics had a beneficial effect on scores for overall symptoms, abdominal pain, bloating, and flatulence (128). However, the effects may differ between IBS subtypes, such as IBS-D and IBS-C. A study by Chen et al. conducted a three-level meta-analysis of 72 randomized controlled trials involving 8,581 participants to summarize the therapeutic effects of probiotics on IBS (129). The results showed that probiotics significantly outperformed placebo in improving overall IBS symptoms, abdominal pain, and quality of life, though there was notable heterogeneity. Additionally, treatment duration was inversely related to effectiveness, with treatments lasting 4 weeks showing better results, and probiotic strains of *Bacillus* and *Bifidobacterium* were more effective than yeast strains, with *Bacillus* showing superior improvement in abdominal pain. A 2024 meta-analysis reviewed 20 RCTs with 3,011 patients (130). It found that probiotics improved global IBS symptoms better than placebo (RR 1.401, 95% CI 1.182–1.662). They also enhanced quality of life. For relieving abdominal pain, shorter treatments (<8 weeks) and high-dose or multi-strain formulas were more effective. Adverse events did not increase. However, there was high heterogeneity across studies. This suggests a need for larger, standardized trials.

A study by Barbaro et al. explored the effects of a probiotic mixture consisting of two *Lactobacillus* strains (CECT7484 and CECT7485) and one *Lactococcus* strain (CECT7483) on restoring IBS-related increased intestinal permeability, revealing that the probiotics significantly reduced paracellular permeability by upregulating β-actin expression (131). Additionally, high doses of the probiotic mixture increased CYP1A1 expression and produced large amounts of indole-3-lactic acid, suggesting a potential metabolic mechanism that may contribute to its therapeutic effects in IBS. *Akkermansia muciniphila* is a next-generation probiotic. This bacterium is known for its ability to degrade mucus, which is a key component of the gut lining. A study by Meynier et al. demonstrated that inactivated *Akkermansia muciniphila* improves IBS-like symptoms in mice by reducing colonic hypersensitivity, enhancing intestinal barrier function, and increasing IL-22 levels. Additionally, inactivated *Akkermansia muciniphila* alleviates anxiety-like behaviors and memory deficits in a *Citrobacter rodentium* infection model (132). The mechanisms underlying these effects may be related to the inhibition of neural cell responses induced by capsaicin and an inflammatory soup, as well as the anti-hyperalgesic and neuroinhibitory properties of the bacteria. In studies targeting SIBO-related IBS, changes in methane or hydrogen production after probiotic supplementation suggest that probiotics may play a role in regulating small intestine microbiota, though more targeted trials are needed to confirm these findings (133–135).

Recent meta-analyses indicate that any benefit of probiotics in IBS is strain- and combination-specific, with an overall low to very low certainty of evidence by GRADE; consequently, major guidelines (e.g., ACG) suggest against their routine use for global IBS symptoms (136, 137). Across >7,000 participants in 55 RCTs, the relative risk of any adverse event was not increased versus placebo, but adverse-event reporting is inconsistently captured and often under-classified, limiting firm safety conclusions (137). However, given the overall safety of probiotics, their use may still be considered on an individual basis. Going forward, trials should mandate systematic adverse event (AE) documentation, classify and grade events (e.g., CTCAE-aligned), and report post-treatment events transparently to enable robust risk–benefit assessments in IBS.

Prebiotics, as indigestible dietary components, primarily work by providing nutrients to beneficial gut bacteria, thereby indirectly promoting the growth and metabolism of these bacteria and improving gut microbiota structure (138). Common prebiotics include inulin, fructooligosaccharides (FOS), and galactooligosaccharides (GOS), which not only promote the proliferation of *Bifidobacterium* and *Lactobacillus* but also yield SCFAs through fermentation. These metabolic products play key roles in maintaining the acid-base balance in the gut, improving gut motility, and modulating the immune system (139, 140). Studies on prebiotics in IBS show dose-dependent effects. In a study by Silk et al., IBS patients were divided into groups receiving either 3.5 grams or 7 grams of GOS. The results showed that both doses increased the relative abundance of *Bifidobacterium* in stool samples, with the lower dose group showing more significant symptom improvement, while the higher dose group saw increased bloating in some patients (141). A 2025 single-blind RCT assessed an inulin/FOS mixture (9.2 g/day) in 34 patients with IBS-C (142). After 8 weeks, the treatment group showed significant improvements. Quality of life scores (IBS-QoL) rose from 61.0 to 77.4 (*P* < 0.006), while symptom severity scores (IBS-SSS) dropped from 267.3 to 195.8 (*P* < 0.026). Constipation and psychological well-being also improved significantly. This suggests that fermentable fibers like inulin may be especially beneficial for IBS-C patients, likely through modulation of the gut microbiota and the gut-brain axis. When combined, prebiotics and probiotics form synbiotics. These create a synergistic effect, enhancing the colonization and metabolic activity of beneficial bacteria and potentially improving microbiota diversity for better therapeutic outcomes (143). However, current research on synbiotics in IBS treatment is still preliminary, and significant heterogeneity exists between studies, with their long-term safety and optimal dosage requiring further investigation.

### 5.2 Antibiotic treatment

Antibiotics, as a treatment that directly modulates gut microbiota composition, have garnered increasing attention in the treatment of IBS. Non-absorbable antibiotic rifaximin, due to its high local concentration in the gut and minimal systemic absorption, has become one of the first-line treatments for IBS-D (144). In large multicenter RCTs such as TARGET 1 and TARGET 2, IBS-D patients treated with 550 mg rifaximin three times daily for 14 days showed symptom relief rates of 40.7% and 31.7% within a month, which were significantly different from the placebo group *P* < 0.001) (145). A meta-analysis also indicated that rifaximin treatment reduced the relative risk of symptom persistence in IBS patients to 0.84 (95% CI 0.79–0.90), proving that it can significantly improve overall symptoms in the short term (146). Furthermore, rifaximin not only improves abdominal pain and bloating but also has a positive effect on stool consistency. During treatment, the relative abundance of *Bifidobacterium* in patients’ stool increased, while gas-producing bacteria like *Escherichia coli* decreased (147). This reshaping of the microbiota might be one of the key mechanisms for its anti-inflammatory effects, improving gut barrier function, and reducing visceral hypersensitivity. However, some studies have found that after rifaximin treatment, the relative abundance of some bacterial groups, such as *Enterococcus*, *Veillonella*, and *Enterobacteriaceae*, significantly decreased (148). Yet, these changes did not persist after the follow-up period, indicating that its effect on microbiota regulation may be temporary. Additionally, some IBS patients experience symptom relapse after an average of 10 weeks of rifaximin treatment (149). Therefore, repeated treatments may be required to prolong the remission period, and further studies are needed to determine its long-term effectiveness. To enhance efficacy, a 2025 randomized controlled trial compared rifaximin monotherapy with a combination of rifaximin and a multi-strain probiotic (150). The study included 70 IBS patients, and the results showed that the combination therapy achieved symptom relief rates of 65.7% at weeks 4 and 8, significantly higher than the monotherapy group (31.4%, *P* = 0.004). The rate of improvement in quality of life was also markedly higher (65.7% *vs* 37.1% and 34.2%, with *P*-values of 0.017 and 0.009, respectively). The authors noted that the synergistic effect of rifaximin and probiotics might enhance efficacy, but long-term follow-up and mechanistic studies are still needed for further validation.

### 5.3 Fecal microbiota transplantation

Fecal microbiota transplantation (FMT) has gained attention as a microbiome-based therapeutic modality in recent years. The core concept is to restore a balanced gut microbiota by transplanting processed fecal microbiota from healthy donors into the patient’s gut. Initially approved for treating recurrent *Clostridioides difficile* infections, FMT is still in the exploratory phase for IBS treatment, with clinical studies showing mixed results (151–153). It is noteworthy that FMT has a more established, albeit still evolving, role in treating IBD. Multiple studies have shown that FMT is significantly superior to placebo in inducing clinical and endoscopic remission in patients with mild-to-moderate ulcerative colitis (154, 155). The success of FMT in IBD provides a strong rationale for exploring this therapy in IBS, which also involves dysbiosis. Several RCTs and systematic reviews have shown that FMT can improve overall symptoms, bloating, abdominal pain, and quality of life in IBS patients, with some patients experiencing relief lasting months or even years (156–158). In a double-blind placebo-controlled trial by El-Salhy et al., 164 IBS patients were treated with 30g or 60g of donor stool or their own stool (as a placebo) via endoscopic injection into the upper gastrointestinal tract (156). The results showed that after 3 months, the FMT group had a significantly higher symptom relief rate compared to the placebo group. Moreover, FMT was more effective for IBS-D patients than IBS-C patients. Another RCT by Johnsen et al. showed that FMT through colonoscopy significantly reduced IBS-SSS after 3 months, with a symptom relief rate of 65%, compared to 43% in the placebo group (158). In the 12-month follow-up, 56% of patients in the active treatment group maintained a persistent response, while only 36% in the placebo group did so (*P* = 0.075). Notably, studies have found that the abundance of specific microbiota in donor stool, such as *Bifidobacterium*, may serve as a potential biomarker for predicting FMT treatment success, though this marker has yet to be sufficiently validated (159). Notably, a 2025 double-blind randomized trial compared the effects of capsule FMT, enema FMT, and placebo on IBS symptoms (160). In this trial of 45 patients, both capsule and enema FMT significantly reduced IBS-SSS and improved quality of life at 4 weeks. The corresponding clinical response rates were 86.7% and 73.3% respectively, both significantly higher than the placebo group (26.7%). Adverse events for both FMT methods were mild and did not differ significantly. Although the sample size was small, this study suggests that optimizing the FMT delivery method (capsule or enema) may improve clinical outcomes, a finding that requires validation in larger, multicenter trials.

While data from FMT in IBS treatment show some positive signals, many studies report that its efficacy is less than expected. A 2024 meta-analysis of 10 RCTs (involving 573 patients) found no significant difference between FMT and placebo for short-term symptom improvement (161). Likewise, no significant differences were observed in long-term (24–54 weeks) IBS symptoms or severity. The only benefit was a modest short-term improvement in quality of life. The researchers concluded that the current evidence is insufficient to support the use of FMT for IBS in routine clinical practice, highlighting the need to identify which patient populations might benefit and to establish standardized protocols. Heterogeneity in donor selection (including potential ‘super-donor’ effects), delivery route/dose, antibiotic preconditioning, and baseline microbiome/transit likely contributes to variable outcomes. Using a GRADE framework, the certainty of evidence for global IBS symptom improvement is considered low to very low because of imprecision, risk of bias, and inconsistency across trials. Accordingly, we avoid global recommendations and emphasize patient selection and endpoints aligned with subtype/mechanism. Safety signals in RCTs are generally acceptable, but adverse-event capture and classification remain suboptimal; future studies should mandate standardized AE reporting (e.g., CTCAE-aligned) with long-term follow-up and stringent donor screening per current guidance (162).

### 5.4 Dietary interventions

Dietary interventions have gained significant attention as an accessible and non-pharmacological strategy in the management of IBS. FODMAPs refer to a group of short-chain carbohydrates that are poorly absorbed in the small intestine, including fermentable oligosaccharides, disaccharides, monosaccharides, and polyols (163). The high osmolarity and fermentation of these compounds in the colon lead to gas production, which is one of the main causes of symptoms such as bloating, abdominal pain, diarrhea, and constipation (164). Numerous randomized controlled trials and meta-analyses have demonstrated that a low-FODMAP diet significantly improves IBS symptoms, particularly abdominal bloating, abdominal pain, and quality of life. A recent blinded, randomized reintroduction RCT (2024) further confirmed this observation: among 117 patients, 80% showed significant symptom improvement after 6 weeks on a low-FODMAP diet (165). During the subsequent 9-week blinded reintroduction phase, 85% of patients experienced a symptom relapse, triggered by an average of 2–3 types of FODMAPs per patient, with fructans and mannitol being the most common. The trial highlights the importance of identifying individualized triggers. A meta-analysis by Marsh et al. of six RCTs found that a low-FODMAP diet reduced IBS symptom severity scores and improved patients’ quality of life (166). Additionally, an analysis of 10 studies by Varjú et al. supports the advantages of a low-FODMAP diet in relieving overall symptoms (167). Studies comparing a low-FODMAP diet with other dietary interventions, such as a low-lactose diet or the modified NICE diet, show that the low-FODMAP diet has a more prominent advantage in alleviating abdominal pain and bloating (168). However, it should be noted that a low-FODMAP diet may lead to insufficient fiber intake, which could exacerbate constipation in some IBS-C patients, so individual adjustments are necessary.

In recent years, some studies have explored the relationship between the low-FODMAP diet and changes in the gut microbiota. In subjects whose symptoms improved on the low-FODMAP diet, higher levels of specific microbiota such as Bacteroides, Firmicutes, and *Prevotella* were observed, which are associated with increased carbohydrate metabolism (169). However, some studies suggest that a low-FODMAP diet may reduce the levels of *Bifidobacterium* and butyrate, potentially having adverse effects on gut ecology (170, 171). Simultaneously, the addition of probiotics and prebiotics (such as fructooligosaccharides, but not B-GOS) could reverse these changes (172, 173). Therefore, further research is needed to assess the long-term effects of the low-FODMAP diet on gut microbiota and its impact on IBS symptoms. To further explore this synergy, a double-blind randomized controlled trial (n=85) reported on the comparative efficacy of combining a low-FODMAP diet with probiotics (174). The study divided patients into a low-FODMAP diet + probiotic group and a low-FODMAP diet + placebo group. After 3 weeks, both groups showed significant decreases in IBS-SSS and VAS scores, with over 85% of patients experiencing an IBS-SSS reduction of more than 50 points, suggesting that the low-FODMAP diet itself has a substantial effect on symptom improvement. Notably, the probiotic group showed a slight advantage in improving stool form: for IBS-C patients, the proportion of normal stools was 70.6% versus 35.3% in the placebo group; for IBS-D patients, these proportions were 75.0% and 58.8%, respectively. No serious adverse events occurred. Overall, the low-FODMAP diet remains the core intervention, and probiotics may offer an additional benefit in modulating stool form, though this requires validation in larger trials with long-term follow-up.

A gluten-free diet is primarily recommended for IBS patients who are either self-reported or confirmed to be sensitive to gluten, after celiac disease has been ruled out (175). Recent studies have further clarified the biological basis for why gluten-containing wheat products exacerbate symptoms in some IBS patients. Recent *in vitro* and organoid studies have shown that pepsin-trypsin digested α-gliadin, a component of gluten, can bind to the chemokine receptor CXCR3 on intestinal epithelial cells. This activates PLC/IP_3_ signaling, induces calcium release from the endoplasmic reticulum, and triggers the disassembly of tight junctions, thereby increasing intestinal barrier permeability. This process is accompanied by elevated zonulin levels, suggesting that gluten peptides directly interfere with epithelial structure (176). Furthermore, animal and human cell experiments have demonstrated that α-amylase/trypsin inhibitors (ATIs) in wheat are potent innate immune activators, with their content being significantly higher in modern wheat compared to ancient varieties. ATIs are resistant to heat and digestive enzymes, and upon ingestion, they can activate the TLR4–MD2–CD14 complex, leading to the infiltration of intestinal macrophages and dendritic cells and the release of mediators like TNF-α and IL-1β, which induces an inflammatory response that is most pronounced in the colon and decreases progressively through the ileum to the duodenum (177). In *Tlr4*-deficient mice, ATIs no longer induce inflammation, further confirming this pathway (178). Additionally, gluten can contribute to microbial imbalance. A randomized crossover dietary study comparing high-gluten and low-gluten diets in healthy adults found that the low-gluten diet significantly reduced four *Bifidobacterium* species and two butyrate-producing bacteria (*Anaerostipes hadrus* and *Eubacterium hallii*), while certain unclassified members of the *Clostridiales* order and *Lachnospiraceae* family increased, indicating that reducing gluten intake alters carbohydrate metabolism pathways (179). It is important to note that fructans (a type of FODMAP) and ATIs, which are abundant in wheat, may trigger symptoms independently of gluten itself; double-blind challenge trials have shown that in individuals who self-report “gluten sensitivity,” fructans are often the primary symptom trigger (180). Therefore, based on these mechanisms, dietary adjustments for IBS patients should consider gluten, FODMAPs, and individual microbial characteristics, and should be validated through individualized trials.

High-fiber diets are especially suitable for IBS-C patients. Fiber can be classified into soluble and insoluble types, with soluble fibers (e.g., psyllium husk, inulin) improving stool consistency, increasing stool volume, and promoting gut motility (181, 182). Additionally, soluble fibers (e.g., inulin and fructooligosaccharides) are primarily used as energy sources by the gut microbiota, promoting the growth of beneficial bacteria such as *Lactobacillus* and *Bifidobacterium* (183). Dietary supplementation with soluble fiber has been associated with positive changes in the gut microbiota composition. Studies have shown that after 7 days of psyllium supplementation, beneficial microbes like *Faecalibacterium*, Bacteroides, and *Roseburia* significantly increased in IBS-C subjects. These bacteria are associated with the production of SCFAs like butyrate and increased stool water absorption (181). A study by Wang et al. used food frequency questionnaires and fecal metagenomic data from 969 participants aged 18–65 to investigate dietary risk factors and gut microbiota interactions in IBS subtypes (184). Compared to non-IBS individuals, IBS-D patients consumed more healthy plant-based foods and fiber, while IBS-C patients tended to consume more unhealthy plant-based foods. The study also found that IBS-D patients exhibited lower microbial diversity and a reduction in strict anaerobes such as *Prevotella copri*, while IBS-C patients showed a slight increase in pro-inflammatory microbiota. In individuals with higher *Prevotella copri* abundance, fiber and iron intake were more strongly and positively correlated with IBS-D. Some studies suggest that switching from a high-fiber to a low-fiber diet can quickly worsen IBS symptoms (185, 186), indicating that adequate and balanced fiber intake is crucial for maintaining gut function. In contrast to soluble fiber, insoluble fiber (e.g., wheat bran) does not dissolve readily in water. It primarily shortens colonic transit time by absorbing water to increase fecal volume and by providing mechanical stimulation to the colonic mucosa. Numerous studies and recent guidelines indicate that while this mechanical stimulation can increase defecation frequency, it does not significantly improve global IBS symptoms and may even exacerbate bloating, gas, and abdominal pain upon initial intake (187, 188). Therefore, insoluble fiber is not a universal choice for all IBS patients and should be used with caution, especially in those with diarrhea. However, the impact of insoluble fiber on the gut micro-ecology is gaining attention. A double-blind randomized controlled trial that divided healthy subjects into four groups with or without wheat bran (WB) and barley (BM) for a 4-week intervention found that the WB intake group had significantly higher fecal butyrate concentrations and a greater abundance of butyrate-producing bacteria (such as *Ruminococcus*, *Faecalibacterium*, and *Roseburia*) compared to the non-WB group. When WB was combined with barley rich in β-glucans, the relative abundance of the Bacteroides genus increased significantly. This study suggests that insoluble fiber may enhance short-chain fatty acid production and potentially improve gut barrier function by promoting the proliferation of butyrate-producing flora and Bacteroides (189). Therefore, in the dietary management of IBS, the choice of insoluble fiber requires balancing its potential negative impact on symptoms against its possible benefits for the gut microbiota. For patients with constipation, certain non-fermentable or low-fermentability insoluble fibers (such as cellulose, guar gum, etc.) can be gradually introduced under professional guidance while monitoring symptoms and microbial changes. Future randomized controlled trials and molecular-level research are needed to clarify the safety, efficacy, and micro-ecological regulatory mechanisms of insoluble fiber in different IBS subtypes.

### 5.5 Novel treatments

With the rapid development of molecular biology, metabolomics, and synthetic biology, microbiome-based IBS treatments are evolving from traditional probiotics, prebiotics, antibiotics, and FMT to more cautiously explored, hypothesis-driven and mechanism-informed strategies. An overview of these emerging strategies, including their mechanisms of action, primary study designs, current stage of research, and key challenges, is detailed in **Table 6**.

**Table 6:** Summary of novel treatment strategies for IBS.

Treatment strategy	Key mechanism	Evidence setting	Study design	Key challenges & future directions
SCFA supplementation/targeted agonists	Enhance barrier function, reduce inflammation, provide energy to colonocytes.	Preclinical/Early Clinical	*In vitro* cell models, animal models (e.g., colitis models), small-scale human pilot studies.	Bioavailability, targeted delivery (e.g., microencapsulation), optimal dosing, long-term safety. Requires well-designed RCTs.
Engineered probiotics (gene-edited)	*In-situ* production of anti-inflammatory molecules (e.g., IL-10), targeted removal of pathogens, enhanced colonization.	Preclinical	*In vitro* co-culture systems, animal models (mice, pigs).	Off-target effects, horizontal gene transfer, biocontainment, manufacturing standardization, regulatory approval. Requires rigorous safety and efficacy testing in humans.
Postbiotics	Modulate immune responses, enhance barrier integrity, direct antimicrobial effects. More stable than live probiotics.	Preclinical/Exploratory Clinical	Ex vivo organoid/tissue models, animal studies, a few small, unblinded human trials.	Heterogeneity of preparations, lack of standardized production, dose-finding. Requires large, multi-center RCTs to confirm efficacy.
Gut-brain axis modulators	Regulate visceral hypersensitivity, motility, and mood by targeting neural receptors (e.g., opioid, cannabinoid) influenced by microbiota.	Clinical (Phase II/III/IV)	Randomized, placebo-controlled clinical trials (RCTs).	Balancing efficacy with side effects (e.g., constipation, pancreatitis risk), identifying patient subgroups most likely to respond.
Personalized treatment (AI & multi-omics)	Use individual patient data (microbiome, metabolome, etc.) to predict optimal treatment (diet, probiotics, etc.).	Exploratory/Early Clinical	Retrospective cohort analyses, prospective observational studies, some pilot RCTs.	Requires external validation of predictive models, high cost, integration into clinical workflow. Needs pragmatic RCTs to prove superiority over standard care.

Novel treatment strategies encompass several aspects: on one hand, they involve regulating gut microbiota metabolic products, such as the exogenous supplementation of SCFAs or the development of targeted agonists, to improve gut barrier function and reduce inflammation. For example, butyrate can not only inhibit the NF-κB signaling pathway and reduce pro-inflammatory cytokine secretion but also exert anti-inflammatory and regulatory effects by modulating receptors such as GPR43, GPR41, and GPR109A (190). Accordingly, these approaches should be regarded as experimental; any direct SCFA supplementation, targeted agonists, or formulation technologies (e.g., microencapsulation) should be evaluated in well-designed, placebo-controlled trials with standardized endpoints and adverse event grading, before clinical adoption. Additionally, advances in metabolomics allow for more precise analysis of specific metabolic deficiencies or excesses in IBS patients, providing a basis for hypothesis generation and target prioritization rather than immediate routine use.

On the other hand, gene-editing technologies (such as CRISPR-Cas9) can be used to engineer probiotics, improving their resistance to stomach acid, tolerance to bile, and colonization ability, as well as enabling them to monitor intestinal inflammation and secrete anti-inflammatory cytokines. For instance, *Lactobacillus rhamnosus* engineered to secrete IL-10 or other anti-inflammatory factors upon detecting local inflammation has been demonstrated primarily in preclinical systems (191). At present, evidence in IBS is limited and of low-certainty; potential risks include off-target effects, horizontal gene transfer, uncontrolled colonization/durability, and challenges in manufacturing standardization and regulatory/biocontainment oversight. Gene editing could also theoretically be used to reduce the abundance of methane-producing archaea like *Methanobrevibacter smithii* in IBS patients as a theoretical approach; any such strategy requires rigorous human testing with safety monitoring. Furthermore, synthetic biology can design artificial microbiota communities, combining multiple optimized strains to reconstruct a healthy, stable gut ecosystem, which is especially important for IBS patients with severe dysbiosis. Findings from animal/ex vivo models are encouraging but remain insufficient for routine clinical use; we frame these as future directions pending adequately powered, placebo-controlled trials with pre-specified safety oversight.

Moreover, postbiotics (i.e., non-living bioactive substances secreted by probiotics) represent a promising yet still exploratory treatment approach with potential advantages in stability. Existing research has shown that postbiotics may regulate inflammation, enhance epithelial barrier function, and modulate gut immunity (192). A recent study found that Lactobacillus casei LC-DG and its postbiotics reduced inflammatory readouts in ex vivo systems and modulated cytokine profiles (193). However, clinical evidence in IBS remains limited, with heterogeneity in preparations, dosing, and outcome measures; claims of superior safety/effectiveness should await standardized production, quality control, and trial-level AE capture/CTCAE-aligned grading. Future work should prioritize manufacturing standardization, dose-finding, and multi-center RCTs to determine efficacy and safety profiles in specific IBS subtypes.

In addition, drug development targeting the gut-brain axis is in the exploratory phase. Novel drugs could improve gut motility and regulate neurotransmitter release by modulating the vagus nerve, opioid receptors, and cannabinoid receptors, thereby alleviating IBS symptoms while improving the patient’s psychological state. For example, Eluxadoline (Viberzi, Allergan), a mixed opioid receptor modulator, has shown modest efficacy in alleviating overall IBS symptoms and improving quality of life in certain clinical trials (194). It shows context-dependent effectiveness relative to rifaximin, but its side effects (such as constipation, abdominal pain, and the risk of pancreatitis) require careful patient selection and monitoring (195). Future studies should adopt subtype-specific enrichment and pre-specified safety thresholds.

Furthermore, with the rapid development of high-throughput sequencing and artificial intelligence, personalized treatment is becoming increasingly feasible in principle. By conducting comprehensive multi-omics analyses (including genomics, microbiomics, metabolomics, and epigenomics) of IBS patients, we can reveal differences in microbiota structure and functionality among different patients. Machine learning algorithms can then be used to establish predictive models, accurately selecting the most appropriate treatment strategies for each patient. At present, predictive models require external validation, calibration, and assessment for overfitting/confounding; studies have shown that patients respond differently to probiotics, prebiotics, and even FMT, so personalized intervention plans based on the patient’s microbiota profile should be tested prospectively in pragmatic RCTs rather than assumed to be effective. This data-driven precision medicine model may optimize treatment selection for subsets of patients, providing testable hypotheses for long-term management of IBS.

Novel treatment strategies offer several potential breakthroughs in microbiome-based interventions for IBS management. By systematically intervening from multiple angles, such as microbiota metabolic regulation, gene engineering, postbiotics development, gut-brain axis modulation, and personalized precision medicine, these new approaches can address the limitations of traditional probiotic and antibiotic treatments and provide new possibilities for long-term, stable, and personalized IBS therapy. Future research should verify the efficacy of these novel methods through large-scale, multi-center, randomized, double-blind, and long-term follow-up RCTs, while utilizing multi-omics and big data technologies to delve into their mechanisms of action, ultimately achieving the goal of comprehensive treatment based on microbiome-based precision regulation.

## 6 Conclusion and outlook

IBS is a common disorder of gut–brain interaction in which gut microbiota dysbiosis is associated with symptoms rather than being uniformly causal. Converging data indicate that microbial functions—particularly the metabolism of short-chain fatty acids, bile acids, gases, and tryptophan-derived products—can influence epithelial barrier integrity, mucosal immune tone, motility, and gut–brain signaling, thereby modulating IBS symptoms. While dysbiosis is a common pathophysiological link, the microbial shifts and resulting low-grade inflammation in IBS are subtler than the pronounced dysbiosis and overt inflammation characteristic of IBD, a distinction that is critical for developing targeted therapies. Subtype-aware patterns are emerging (e.g., primary bile acids and faster transit in IBS-D; methanogenesis and slower transit in IBS-C), yet effect directions and magnitudes differ across cohorts, and individual treatment response remains variable. Accordingly, this narrative review synthesizes taxonomy-to-function links and appraises microbiome-related interventions with attention to efficacy, safety, and the certainty of evidence.

However, a critical appraisal reveals significant drawbacks in the existing body of literature, highlighting major research gaps. First, most observational studies are cross-sectional snapshots, incapable of capturing the dynamic nature of the microbiota or establishing causality. They are often small-scale and poorly controlled for profound confounders like diet and medication, leading to inconsistent and non-reproducible taxonomic findings. Second, methodologically, reliance on fecal samples may miss key mucosal interactions, and differences in sequencing and analysis pipelines severely hamper cross-study comparability. Third, functionally, the field remains largely taxonomy-centric; multi-omics data linking specific microbial metabolic outputs to host pathophysiological changes in humans are still scarce. Finally, regarding interventions, clinical trials are frequently plagued by high heterogeneity, small sample sizes, short durations, and a lack of standardized adverse event reporting, resulting in a low certainty of evidence for most therapies and an inability to guide personalized care.

To bridge these gaps, priorities for future work must include: (i) longitudinal, subtype-stratified multi-omics studies with repeated sampling to define temporal trajectories linking microbial functions to symptom flares; (ii) biomarker-enriched, mechanism-aligned trials (e.g., bile-acid–targeted therapy for suspected bile-acid malabsorption in IBS-D); (iii) large-scale pragmatic RCTs with pre-registered protocols, harmonized core outcomes, and transparent reporting of negative results; and (iv) standardized, end-to-end methodological protocols (from sampling to analysis) to ensure reproducibility. Emerging approaches—such as postbiotics, engineered biotherapeutics, and AI-driven personalized medicine—remain investigational and must proceed through rigorous, well-powered studies with long-term safety monitoring. Through these concerted efforts, microbiome-informed strategies may evolve from blunt tools to precision instruments, ultimately improving symptom control and quality of life for people with IBS.
